# Chronic compression induces transcriptional, metabolic, and functional state changes in macrophages that recapitulate tumor-associated phenotypes

**DOI:** 10.3389/fimmu.2025.1626024

**Published:** 2025-12-09

**Authors:** Alice Burchett Darantiere, Hao Chen, Julian Najera, Scott Howard, Meenal Datta

**Affiliations:** 1 Department of Aerospace and Mechanical Engineering, University of Notre Dame, Notre Dame, IN, United States; 2 Department of Electrical Engineering, University of Notre Dame, Notre Dame, IN, United States

**Keywords:** Myeloid cells, solid stress, immunomechanics, mechano-immunology, metabolism, fluorescence lifetime imaging (FLIM), polarization, glioblastoma.

## Abstract

**Introduction:**

Macrophages comprise a significant portion of the glioblastoma tumor microenvironment and are essential in promoting immunosuppression and tumor progression. Solid tumors such as glioblastoma generate solid stress as they expand, creating a compressive microenvironment for mechanosensitive immune cells including macrophages. Macrophages are known to respond to various mechanical stimuli but have not yet been studied in the context of chronic compression observed in growing tumors.

**Methods:**

Here, we used a custom *in vitro* compression system to elucidate the effects of compressive solid stress on murine macrophages.

**Results:**

We found that macrophages have significant morphological, transcriptional, metabolic, and functional responses to compression. These changes corresponded to both canonical pro- and anti-inflammatory macrophage states. The gene expression signatures of compressed macrophages more closely resembled those of glioma-associated macrophages known to be associated with worse patient outcomes.

**Conclusion:**

These results indicate that compression alone, independent from tumor cell-derived biochemical factors, may contribute to the pathological tumor-associated macrophage phenotype. This could represent a vicious cycle of tumor immunomechanics and mechano-immunology. Targeting solid stress in tumors or the response to solid stress by macrophages may interrupt this feedback loop to help normalize the tumor immune microenvironment and improve glioblastoma response to immunotherapy.

## Introduction

Myeloid cells, such as macrophages, accumulate in the glioblastoma (GBM) tumor microenvironment (TME) and can comprise nearly half of the tumor bulk (1, 2). Macrophage phenotype is often represented as ranging from pro-inflammatory (M1-like) to anti-inflammatory (M2-like). However intermediate and overlapping phenotypes are found in both health and disease (3, 4). In GBM, macrophages adopt a pathological, pro-tumor phenotype, which shares some characteristics of M2-like polarized macrophages (2). As orchestrators of the innate and adaptive immune response, these tumor-associated macrophages (TAMs) contribute to tumor growth, invasion, and treatment resistance (5, 6). They are also key players in resistance to immunotherapies, as they recruit regulatory T cells and inhibit the infiltration, proliferation, and function of cytotoxic T cells through the expression of cytokines and immune checkpoints (7). As a result, TAM density is associated with poor prognosis and reduced overall survival in many cancer types (5, 8–10).

The GBM TME also features elevated solid stress, which arises as the tumor expands and pushes against the surrounding tissue (11–13). Our prior *ex vivo* measurements of murine tumors reveal a range of 0.1 to 10 kPa maximum solid stress, with murine GBMs falling in the range of 0.1 to 0.2 kPa (14–16). In GBM patients, we have found that solid stress ranges between 0.01 and 0.6 kPa, depending on invasive versus nodular growth patterns (16, 17). This chronic compression acts on both the tumor itself and the surrounding brain to cause neuronal death and inhibit fluid transport (16, 18). Compression in solid tumors can collapse blood vessels, exacerbating nutrient deprivation and hypoxia which in turn contributes to the highly angiogenic GBM phenotype (19–21). Angiogenesis in GBM relies in part on MMP-mediated extracellular matrix (ECM) remodeling and VEGF signaling, and microvessel density correlates with poorer prognosis (22, 23). ECM remodeling facilitated by myeloid cells also contributes to GBM invasion and tumor expansion (24).

Macrophages are known to respond to mechanical stimuli, with the polarization and phenotype response highly dependent on the physiological context (25, 26). While solid stress is associated with lymphocyte exclusion in tumors, macrophages maintain a presence in both high- and low-stress tumors (27). Their response to mechanical stimuli such as shear stress, tissue viscoelasticity, cyclic compression or stretching, and hydrodynamic pressure changes has been previously studied, particularly in the context of the cardiovascular and skeletomuscular systems (25, 28–31). We recently showed that macrophages themselves generate solid stress *de novo*, causing deformation of a confining substrate, and that 3-D macrophage cultures respond phenotypically to externally applied chronic compression (32). However, there are no other studies to date that characterize the macrophage response to chronic compressive stress in the context of cancer immunology (33–36).

Macrophage function is closely linked to metabolic state. M2-like polarization is accompanied by an increase in oxidative phosphorylation compared to more glycolytic M1-like macrophages (37–39). While TAMs are often observed to rely on glycolysis for energy production, TAMs in hypoxic environments and in the core of GBM tumors have increased oxidative phosphorylation (40–42). Macrophages and GBM cells participate in reciprocal metabolic cross-talk, resulting in a vicious cycle that promotes GBM growth and invasiveness and accumulation of pro-tumor, immunosuppressive TAMs (40). The mechanical properties of the TME are also directly interconnected with metabolism (43). Macrophages within stiffened, fibrotic TMEs upregulate arginase-1 and metabolize arginine, leading to the depletion of arginine in the TME (44). This suppresses anti-tumor T cell activity and leads to the production of pro-tumor metabolites. Because macrophages can themselves generate compressive solid stress (32), there may be an immunomechanical and metabolic feedback loop involving the GBM mechanical TME and tumor-resident macrophages resulting in immunosuppression and tumor progression.

Here, we utilize a 2D mechanical compression model to interrogate macrophage response to chronic solid stress levels previously measured in GBM. Compression induced alterations in macrophage morphology, including increased cell area and more irregular cell shapes. Compressed macrophages also had significantly altered gene expression as assessed by RNA sequencing, upregulating both M1-like and M2-like genes, defying the simplified M1/M2 macrophage polarization spectrum. They also upregulated genes associated with TAMs across a spectrum of tumor types, including glioma and GBM-specific TAMs. Trending upregulation of canonical M1-like and M2-like markers was consistent across varying magnitudes of solid stress, duration of compression, and in normoxic and hypoxic conditions. Fluorescence lifetime imaging (FLIM) showed alterations in cellular metabolism under compression consistent with pro-tumor polarization. Functionally, compressed macrophages were more phagocytic and released more nitric oxide into their media. These results suggest that chronic compression results in a more complex phenotype than is represented on the traditional pro- and anti-inflammatory axis. This also demonstrates that compression alone, in the absence of any cancer-derived factors, may cause macrophages to adopt a TAM-like state, implicating solid stress as a potentially essential driver of tumor immunosuppression and a promising therapeutic target.

## Materials and methods

### Cell culture and compression

RAW264.7 (ATCC TIB-71) murine macrophages and CT-2A murine GBM cells were cultured in Dulbecco’s Modified Eagle Medium (DMEM, Corning, 10-013-CV) supplemented with fetal bovine serum (FBS, Corning, 35-010-CV) and penicillin/streptomycin (Corning, 30-002-CI). Unless otherwise noted, cells were maintained at 37 °C and 5% CO_2_ in a humidified incubator.

We employed a custom weight system to apply uniaxial compressive stress to cell monolayers, as described previously (45). Cells were seeded atop a cell culture insert with 0.4 um pore size (CellQuart, 9300412) and placed in a 6-well plate. Agarose cushions were made from 1% (w/v) agarose (Bio-Rad, 1613100) dissolved in complete culture medium and formed with custom 3-D printed cylindrical molds to match the size of the cell culture insert. These serve as a media reservoir to prevent nutrient deprivation and protect the cells from direct contact with the rigid weight. The agarose cushions were submerged in culture medium and placed in the incubator for 24 hours before use to equilibrate CO_2_ and oxygen within the gels. After cells had adhered to the cell culture insert (overnight), the agarose cushion was placed on top, and a custom 3-D printed PLA weight was placed on top of the agarose. The mass of the weight is such that it applies 0.14 kPa of compressive stress distributed across the insert membrane area, as quantified by dividing the observed mass of the weight by its surface area. This mimics the approximate magnitude (~0.1-0.2 kPa) derived from prior studies measuring solid stress *in situ* in murine GBM tumors (14). For the control condition, only the agarose cushion was placed on the cells.

After the addition of the agarose (control) or agarose + weight (compressed), the cells were placed in a standard incubator for 24 or 48 hours. For the hypoxia condition, the cells were placed in a hypoxia incubator with 1% O_2_ and 5% CO_2_. Conditioned media was collected from the well underneath the cell culture insert after 24 or 48 hours of compression and stored at -80 °C before downstream analysis or treatment. For M1-polarized macrophages, media was supplemented with 200 ng/ml lipopolysaccharide (LPS, Santa Cruz Biotechnology, sc-3535) and 20 ng/ml IFN-γ (BioLegend, 575302). M2-polarized macrophages were treated with 20 ng/ml IL-4 (Pepro-tech, 214-14). For indirect co-culture conditions, one cell type (RAW264.7 or CT-2A) was seeded on top of the porous transwell membrane, and the other cell type was seeded underneath on the surface of the 6-well plate. Compression was applied as described above to the cells in the transwell for 24 hours.

### Immunofluorescence staining and cell morphology analysis

After compression, the cells were fixed with 4% paraformaldehyde (Thermo Scientific, AAJ19943K2) for 20 minutes at room temperature and rinsed with PBS. The membranes were removed from the cell culture inserts and stored in PBS with 0.1% sodium azide (Sigma-Aldrich, S2002). Phase-contrast images of these fixed membranes were acquired on a Leica DMi 1 microscope. The membranes were cut into several pieces using a razor. Single pieces of the membranes were placed into a 12-well plate for staining using conjugated antibodies against YAP (Santa Cruz Biotechnology, sc-376830), TAZ (Santa Cruz Biotechnology, sc-518026), Piezo1 (Proteintech, 15939-1-AP), and DAPI (Sigma-Aldrich, D9542). The stained membranes were mounted onto microscope slides under coverslips using Fluoromount G (Invitrogen, 00-4958-02). Image Z-stacks were acquired using a Nikon AXR confocal microscope, and the maximum intensity projections were used for downstream analysis. The YAP, TAZ, and DAPI channels were merged to obtain images of the entire cell, and images were cropped to regions that did not contain overlapping cells for more accurate segmentation. The cropped images were segmented using CellProfiler, which quantified cell area and shape metrics. Representative images shown were created by staining cells with CellMask actin tracking stain (Invitrogen, A57244) and DAPI, and were enhanced for clearer visualization.

### Fluorescence lifetime imaging

For fluorescence lifetime imaging (FLIM), cells were seeded sparsely in a 12-well cell culture insert and compressed as described above. After 24 or 48 hours, the weight and agarose cushion were carefully removed and the cells were fixed as described above. The membrane was removed from the cell culture insert and mounted using Fluoromount G onto glass microscope slides. The coverslip edges were sealed with nail lacquer to prevent moisture from reaching the membranes. The slides were stored at 4°C before imaging.

FLIM measures the inherent lifetime (τ) of fluorophores regardless of intensity, enabling tracking of bio-microenvironment dynamics. In this study, we utilized a custom-built two-photon excited frequency-domain FLIM system. “Instant-FLIM” captures intensity and lifetime imaging simultaneously under 800 nm excitation for intrinsic fluorescence lifetime through precise measurements of endogenous fluorophores such as NAD(P)H (46). For each measurement, lifetime phasor coordinates g and s [2] are averaged separately to calculate the mean lifetime per field of view (FOV) for quantitative analysis (47).

### Phagocytosis and Griess assay

Cells were either compressed as described above on a transwell insert or cultured in conditioned media in a standard cell culture plate from compressed/control macrophages for 24 hours. After treatment, cells were incubated with 1 µm fluorescent polystyrene beads (Invitrogen, F13083) for 2 hours. The cells were then fixed, stained with CellBrite blue (Biotium, 30024), and imaged. We used CellProfiler to quantify the intensity of red fluorescence within the blue cell area. The conditioned media of compressed macrophages was analyzed using a Griess reagent system following the vendor protocol (Promega, G2930).

### RNA sequencing and bioinformatics analysis

RNA was extracted as described above from six samples each of 24h compressed (0.14 kPa) and 24h agarose-only control macrophages and sequenced using the NovaSeq X Plus 10B flow cell at a depth of approximately 50 million average raw reads per sample. The raw data was aligned to the GENCODE mouse genome annotation using STAR 2.7.2. The resulting expression data was analyzed using DESeq2 to identify differentially expressed genes (DEGs), create the volcano plot, create heatmaps, perform gene ontology and geneset enrichment analyses, and generate log_2_(fold change) values for specific genes. For geneset analysis, genesets were either downloaded from the GSEA online database or obtained from relevant publications (**Supplementary Table S1**). For human genesets, human gene names were converted to their murine orthologs using g:Profiler, omitting genes with no identified murine ortholog. To visualize the degree of overlap between genesets, a heatmap was generated using an online multiple list comparator (molbiotools.com) based on the Jaccard index (number of shared genes divided by total number of genes in the combined set). The protein-protein interaction network was created using STRING-db, filtering for the subset of DEGs with an absolute value of log_2_(fold change) greater than 2 and an adjusted p-value (padj.) less than 0.05. The interactions were further filtered to include only genes with a high confidence interaction score (greater than 0.9) and disconnected nodes were excluded. Violin plots were created using GraphPad Prism (GraphPad Software, San Diego, CA, USA). A table of differentially expressed genes, filtered for p.adj ≤.05 and base mean > 50, is included in **Supplementary Table S2**.

### Reverse-transcription quantitative polymerase chain reaction

After completion of the compression experiment, the agarose cushion and weight were removed. The cells were removed from the membrane of the cell culture insert using a cell scraper. These were pelleted, and the RNA was extracted (Zymo Research, R2051). Gene expression was analyzed using TaqMan primers for *Arg1* (Thermo Fisher Scientific, Mm00475988_m1), *Nos2* (Mm00440502_m1), *Ki67* (Mm01278617_m1), *Casp3* (Mm01195085_m1), *Il1b* (Mm00434228_m1), *Serpinb2* (Mm00440905_m1), *Cd47* (Mm00495011_m1), and *Gapdh* (Mm99999915_g1). The raw PCR data was analyzed using the qPCR Design and Analysis app (Thermo Fisher Scientific). Gene expression was normalized to GAPDH and then normalized to the mean of the control group and reported as 2^− ΔΔCt^. Each condition and gene had between 3 and 6 biological replicates.

### Statistical analysis

Statistical analyses and data visualization were done using GraphPad Prism. Cell size, circularity, solidity, and fluorescence lifetime were compared using a Mann-Whitney test. Phagocytosis fluorescence intensity and nitrite in media were compared using an unpaired t-test. Error bars represent standard error of the mean and asterisks indicate statistical significance (*p ≤ 0.05, **p ≤ 0.01, ***p ≤ 0.001, ****p ≤ 0.0001). For RT-qPCR data, asterisks indicate statistical significance adjusting for multiple comparisons via multiple unpaired t-tests.

## Results

### Compressed macrophages have increased size and irregular morphology

**Figure 1:**
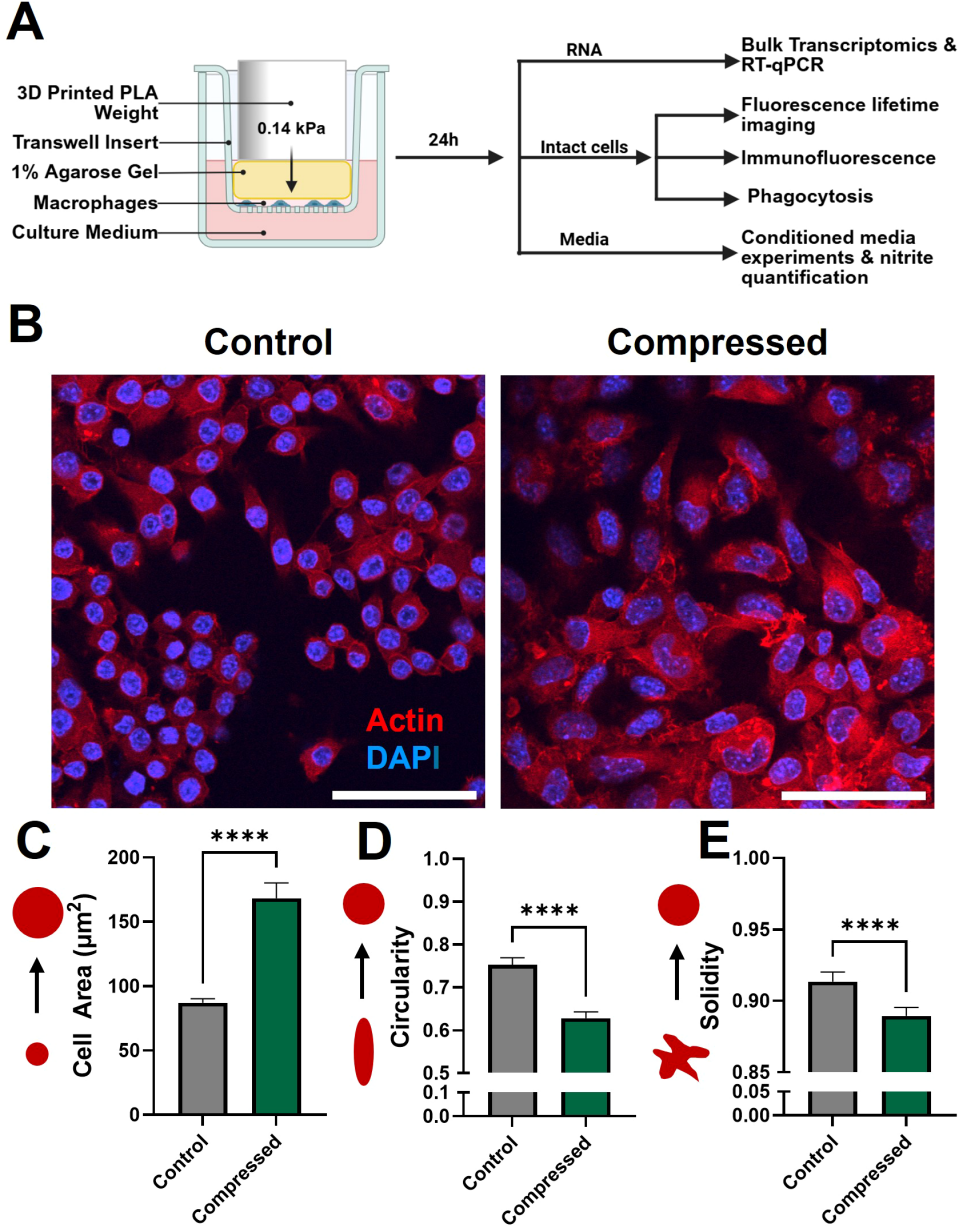
Compressed macrophages have increased size and more irregular morphology. **(A)** Schematic of the macrophage compression system and downstream analyses. Cells are seeded in a monolayer on a porous cell culture insert, and 0.14 kPa compression is added via a 3D-printed weight. The compressed macrophages are either lysed for RNA extraction, or fixed on the membrane for imaging. Conditioned media is collected for nitrite quantification and conditioned media experiments. **(B)** Fluorescence images of control and compressed macrophages stained for actin (red) and nuclei (blue). Scale bar is 50 µm. Quantification of cell area **(C)**, circularity **(D)**, and solidity **(E)**, quantified using an automated CellProfiler pipeline which identifies and characterizes individual cells in fluorescence images. ****p ≤ 0.0001.

Murine RAW264.7 macrophages were first compressed with 0.14 kPa of compressive stress for 24 hours under standard culture conditions. This magnitude of compression approximates the magnitude we previously measured in murine glioblastoma models and in patients (14–17). Both compressed and control macrophages maintain adherence to the surface of the transwell insert, even after removal of the weight and agarose cushion, allowing for downstream staining and morphology analysis. After compression, macrophages were processed for downstream transcriptomic, imaging, and functional assays (**Figure 1A**). Quantification of fluorescent staining (**Figure 1B**) reveals that compression caused cell area to nearly double, from 87.0 µm^2^ to 168.3 µm^2^ (**Figure 1C**). This is consistent with a more pro-inflammatory polarization (48). We also quantified cell circularity, defined in CellProfiler as Form Factor, or 4*π*Area/Perimeter^2^. For a perfect circle, this value equals 1, with smaller values indicating more irregular shapes. Circularity was significantly decreased in the weighted condition (**Figure 1D**). Decreased circularity has been observed with anti-inflammatory, M2-like polarization (48). Solidity is the ratio of the cell area to the convex hull area (the smallest convex shape that would contain the cell) and is a metric that captures irregularity in the cell boundary, distinct from size and circularity. Solidity significantly decreased in compressed macrophages compared to uncompressed controls, reflecting the increased cell boundary irregularity (**Figure 1E**). This is also consistent with anti-inflammatory polarization (48). Polarization of macrophages by cytokine addition has been reported to alter macrophage elongation (49), but compressed macrophages displayed no significant change in elongation or eccentricity (data not shown).

We performed immunofluorescent staining on YAP, TAZ, and Piezo1 to determine if their localization or abundance was altered with compression (**Supplementary Figure S1**). While we observed a statistically significant decrease in the nuclear/cytoplasmic ratio of both YAP and TAZ in compressed macrophages, neither condition had appreciable nuclear signal as would be expected with a biologically significant response (50). There was no significant difference in Piezo1 intensity between compressed and uncompressed macrophages (**Supplementary Figure S1**).

### Compression induces altered expression of genes related to migration, proliferation, and IL-1β signaling

**Figure 2:**
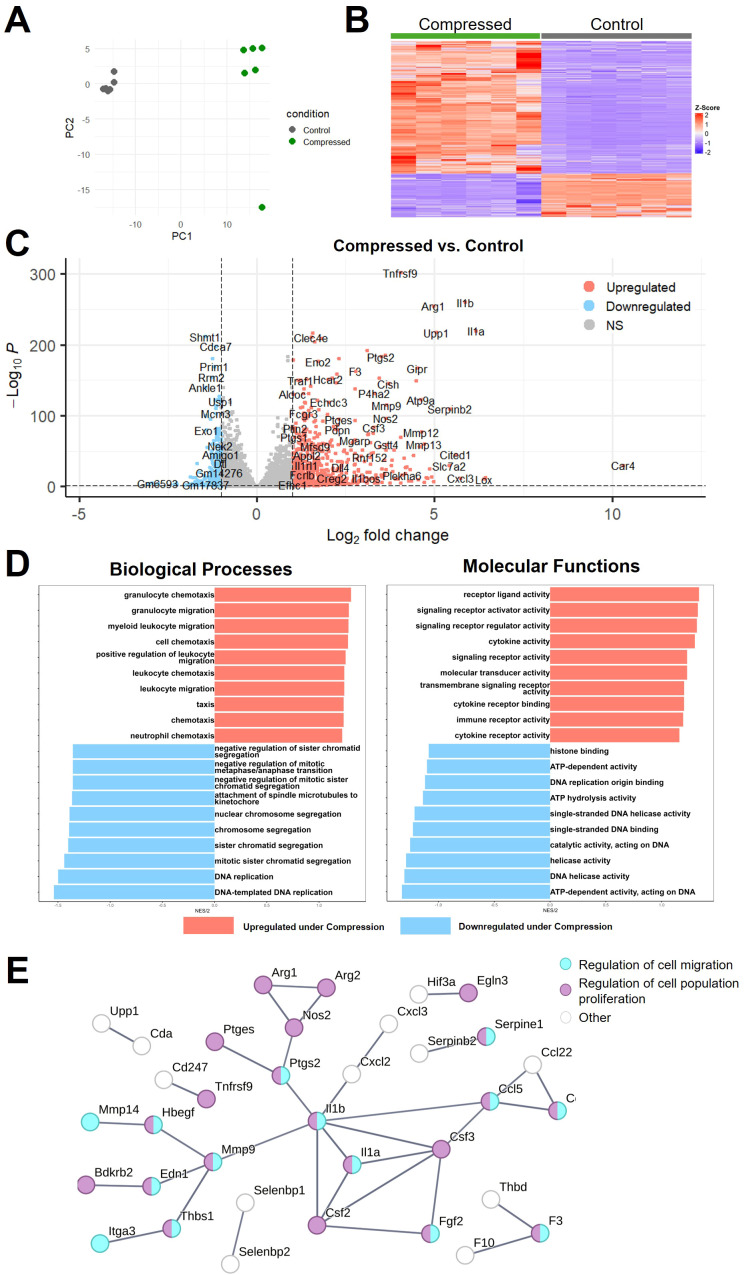
Compression induces changes in gene expression related to migration, proliferation, and IL-1β signaling. **(A)** First and second principal component plots of n=6 control (gray) and compressed (green) macrophage samples. **(B)** Heatmap showing DEGs in compressed versus control samples. **(C)** Volcano plot of the top differentially expressed genes in compressed versus control macrophages, with upregulated genes shown in red, downregulated genes shown in blue, and genes with padj. > 0.05 or absolute value of log_2_(fold change) < 1 shown in grey. **(D)** Gene ontology analysis of the top 10 up- and down-regulated (red and blue, respectively) biological processes and molecular functions in compressed versus control macrophages. **(E)** Protein-protein interaction map showing DEGs with strong interactions, colored based on involvement in regulation of cell migration (cyan) and regulation of cell population (purple).

To determine macrophages’ transcriptional response to chronic compression, we performed bulk RNA sequencing on macrophages after 24 hours of compression compared to uncompressed controls. Compressed macrophage samples clustered separately from control macrophage samples in a principal component analysis (**Figure 2A**) and demonstrated clear differences in gene expression, as shown in a heatmap of differentially expressed genes (DEGs) (**Figure 2B**). There were 7,463 significantly differentially expressed genes (DEGs) (p.adj ≤ 0.05). Of these DEGs, 129 had a log_2_(Fold Change) value below -1, and 319 genes had a log_2_(Fold Change) above 1, as visualized in a volcano plot in **Figure 2C**. We next performed gene ontology analyses to determine which relevant biological and molecular functions were altered (**Figure 2D**). The most upregulated biological processes were mainly related to migration and chemotaxis, behaviors that are more prominent in pro-inflammatory macrophages (51). Relevant significantly upregulated genes include members of the CCL family, including *Ccl2-7, Ccl9*, and *Ccl22* (**Supplementary Table S1**). Downregulated processes were mostly involved in proliferation, an effect observed in macrophages treated with pro-inflammatory stimuli (52). Examples of significantly downregulated proliferation-related genes include *E2f1, E2f2*, and *E2f3* (**Supplementary Table S1**) (53). The molecular functions that were up- and down-regulated corroborate these results, with increased transcription of cytokine and signaling activity, and decreased DNA-related functions (**Figure 2D**).

To visualize the interaction between migration and proliferation-related genes, we generated a protein-protein interaction map (**Figure 2E**). The DEGs involved in migration and proliferation shared many common nodes, with *Il1b* appearing as the most central node. *Il1b* was one of the most highly upregulated genes under compression (**Supplementary Table S2**). TAMs expressing *Il1b* contribute to pathological inflammation, typically expressing inflammatory response genes but failing to generate an effective immune response (54). Increased IL-1β signaling in patients is reported to contribute to GBM growth and stem cell phenotype, and is associated with shorter overall survival (55). *Serpinb2* was also significantly increased, and is considered an M2-like TAM marker that is also implicated in chronic infection (56). We validated that *Il1b* and *Serpinb2*, representative pro- and anti-inflammatory genes, were upregulated via PCR (**Supplementary Figure S2**).

### Compression induces enrichment of polarization and function-related genesets

**Figure 3:**
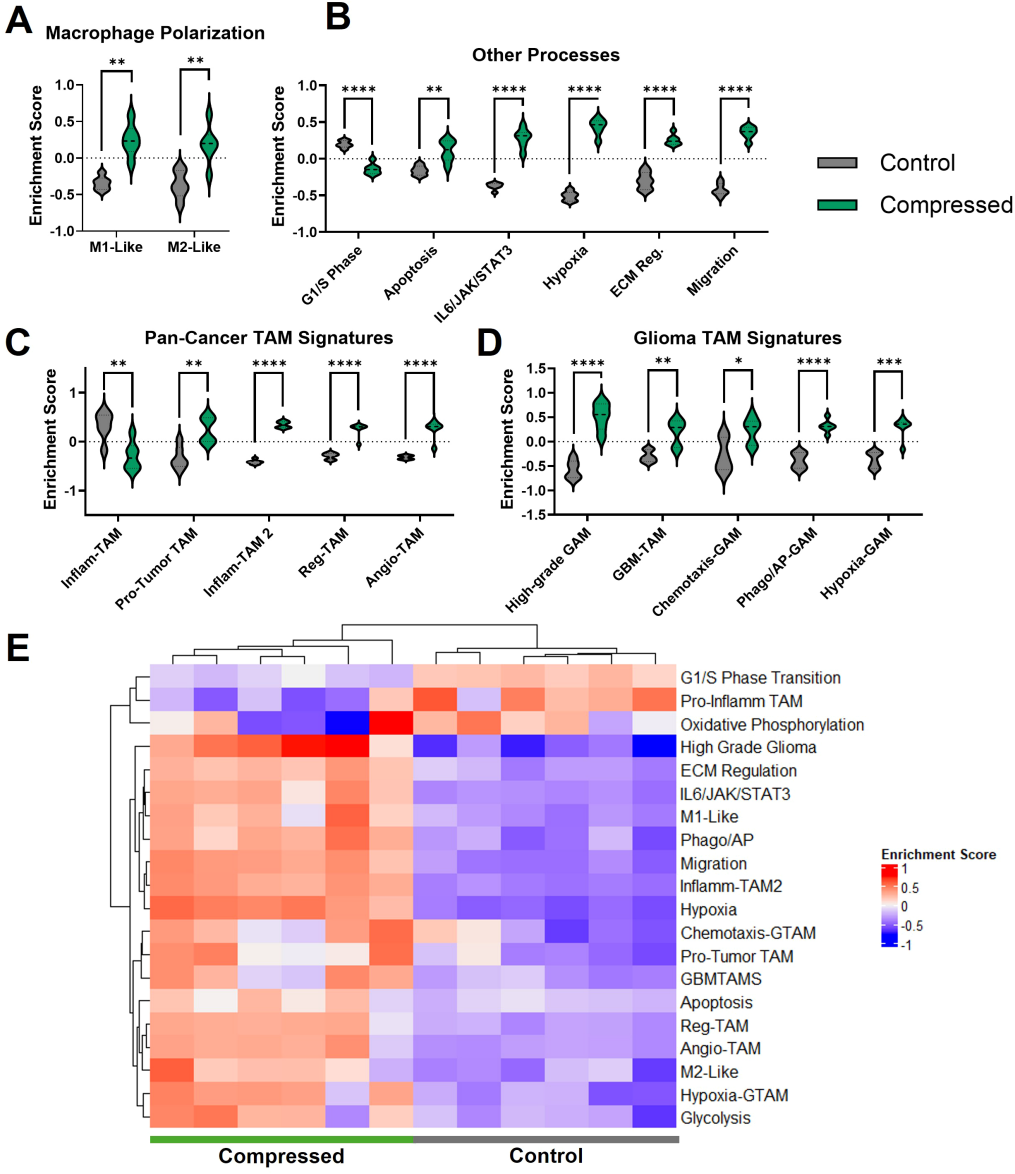
Geneset enrichment analysis reveals enrichment of polarization, functional, and TAM-associated genesets. Enrichment analyses of compressed versus control macrophages for M1-like and M2-like gene sets from literature **(A)**, other biologically relevant pathways from the GSEA online public database **(B)**, tumor-associated macrophage signatures from literature **(C)**, and glioma-specific tumor associated macrophage signatures from literature **(D)**. **(E)** Heatmap of enrichment scores for the gene sets shown above. Asterisks indicate statistical significance based on the p-values adjusted for multiple comparisons (*p adj≤ 0.05, **padj ≤ 0.01, ***padj ≤ 0.001, ****padj ≤ 0.0001).

We next performed geneset variation analysis to determine changes in specific pathways of interest, beginning with macrophage polarization. Although dividing macrophage phenotype into M1-like versus M2-like is an oversimplification of the complex macrophage polarization spectrum, we compared our macrophage expression data to published M1 and M2 genesets to obtain a simplified readout of pro- or anti-inflammatory status. We chose genesets that were created as a result of a meta-analysis of murine macrophage polarization experiments, choosing a geneset that was shared between *in vitro* and *in vivo* M1 and M2 polarized macrophages (57). Compressed macrophages had significant enrichment of both M1-like and M2-like signatures compared to uncompressed controls (**Figure 3A**). Upregulated genes in the M1-like geneset included *Ptgs2*, and M2-related genes included *Jun*, (**Supplementary Table S2**). Other upregulated canonical M1-like polarization markers include *Nos2*, *Il1a* and *Il23a.* Upregulated M2-like markers include *Arg1* and *Tgfb1.*

We conducted analyses on pathways associated with G1-S phase transition, apoptosis, IL-6/JAK/STAT signaling, hypoxia response, ECM regulation, and migration (**Figure 3B**). There was a significant reduction in G1/S phase enrichment with compression, suggesting reduced proliferation. G2, M2M, and general cell cycle genes were also downregulated with compression (data not shown). There was a concurrent modest upregulation of the apoptosis pathway. We also found upregulation of the IL6/JAK/STAT signaling pathway, which is important in regulating macrophage inflammatory response and implicated in cancer immunity dysfunction (58). We observed an upregulation of ECM-modulating genes, also consistent with a migratory phenotype. This included upregulation of *Mmp9, Mmp12, Mmp13, and Mmp14* (**Supplementary Table S2**)*. Mmp9* appeared as a prominent hub in the protein-protein interaction map (**Figure 2E**). There was also an upregulation in hypoxia response genes. It is unclear whether this is in response to hypoxic oxygen levels, or due to mechanically-induced HIF-1α activation as observed in other mechanical settings (59–61). We also observed a significant increase in migration-associated genes. Among these were monocyte chemoattractants such as *Ccl2* and *Ccl5* (**Supplementary Table S2**).

### Compression induces enrichment of genesets associated with pan-cancer TAMs and glioma TAMs

Because the simultaneous upregulation of both M1-associated and M2-associated genes has been observed in tumor-associated macrophages (62), we next tested our expression data against a set of tumor-associated macrophage signatures. First, we compared signatures associated with pro-inflammatory TAMs and pro-tumor TAMs, identified in a pan-cancer analysis of nine different cancer types (esophageal cancer, lung cancer, hepatocellular carcinoma, renal carcinoma, pancreatic cancer, thyroid carcinoma, breast cancer, stomach adenocarcinoma, and colorectal cancer) (63). The pro-inflammatory TAM (Inflam-TAM) signature is reported to be associated with better response to immunotherapy, though both the pro-tumor and pro-inflammatory genesets are reported to be associated with worse prognosis when upregulated in a range of different tumors (63). We found that the Inflam-TAM signature was significantly decreased in compressed macrophages (**Figure 3C**). This signature includes *Gbp3* and *Gbp7*. These guanylate-binding proteins are associated with amplifying the innate inflammatory response against pathogens (64). The pro-tumor signature, in contrast, was significantly upregulated (**Figure 3C**). This genesets includes MMP9, which is reported to promote cancer cell invasion, angiogenesis and disease progression in solid tumors (65).

We then obtained TAM subset gene signatures from a second pan-cancer analysis of 17 tumor types defining inflammatory phenotype macrophages (Inflam-TAM 2), macrophages with an immune-regulatory phenotype (Reg-TAM), and macrophages associated with angiogenesis (Angio-TAM) (66). The upregulated genes from all three of these signatures were significantly upregulated in macrophages under compression (**Figure 3C**). The Inflam-TAM 2 geneset features a range of cytokines that we found to be individually upregulated under compression, including *Il1b, Tnf, Ccl3*, and *Ccl4*. It should be noted that the Inflam-TAM 2 geneset, while labelled inflammatory in the source publication, has similarities to the typical immunosuppressive and tumor-promoting TAM phenotype, such as *Il10ra* (67). The Reg-TAM geneset contained genes including *Lgals3*, which promotes the more aggressive mesenchymal-like state in GBM (68). The Angio-TAM geneset includes genes associated with angiogenesis in cancer, such as *Vegfa*. Inhibition of VEGF, when combined with Ang-2 inhibition, reprograms the immunosuppressive GBM microenvironment and extends survival in animal models (10, 69).

We next explored published genesets for glioma TAMs (GAMs), finding that compression caused significant enrichment of genesets reported to describe several GAM subtypes (**Figure 3D**). Genes associated with macrophages found in the high-grade glioma environment (high-grade GAMs) were enriched upon compression (**Figure 3D**) (70). This includes *Emilin2* and *Gda*. We also analyzed a geneset associated with macrophages in GBM-bearing mice (GBM-TAMs) compared to healthy controls, finding that it was significantly upregulated in compressed macrophages (71). These genes included *Il1b*, highly upregulated under compression as mentioned above, as well as *Ccl5* and *Ptgs2*. Finally, we used the murine orthologs of genesets describing three different GAM subtypes based on analysis of several human gliomas (72). Compressed macrophages had significant enrichment of GAM-specific signatures relating to chemotaxis (Chemotaxis-GAM) and phagocytosis/antigen presentation (Phago/AP-GAM) (**Figure 3D**). Compressed macrophages were also enriched in a GAM signature related to hypoxia response (Hypoxia-GAM), with upregulation of hypoxia-related genes *Adm* and *Bnip3.* Patients with a higher proportion of these Hypoxia-GAMs are reported to have reduced overall survival, and patients with increased *Adm* expression had poorer response to chemotherapy, suggesting a tumor-supportive role (72). A heatmap of the enrichment scores for each pathway demonstrates a clear visual delineation of compressed versus uncompressed conditions (**Figure 3E**). The degree of overlap between the genesets is illustrated in **Supplementary Figure S3**. Most gene sets had relatively few overlapping genes, with the Inflam-TAM 2, Angio-TAM, and Reg-TAM, genesets showing the most overlap.

### Compression induces conserved polarization changes under varying magnitudes and duration of solid stress and exposure to hypoxia

**Figure 4:**
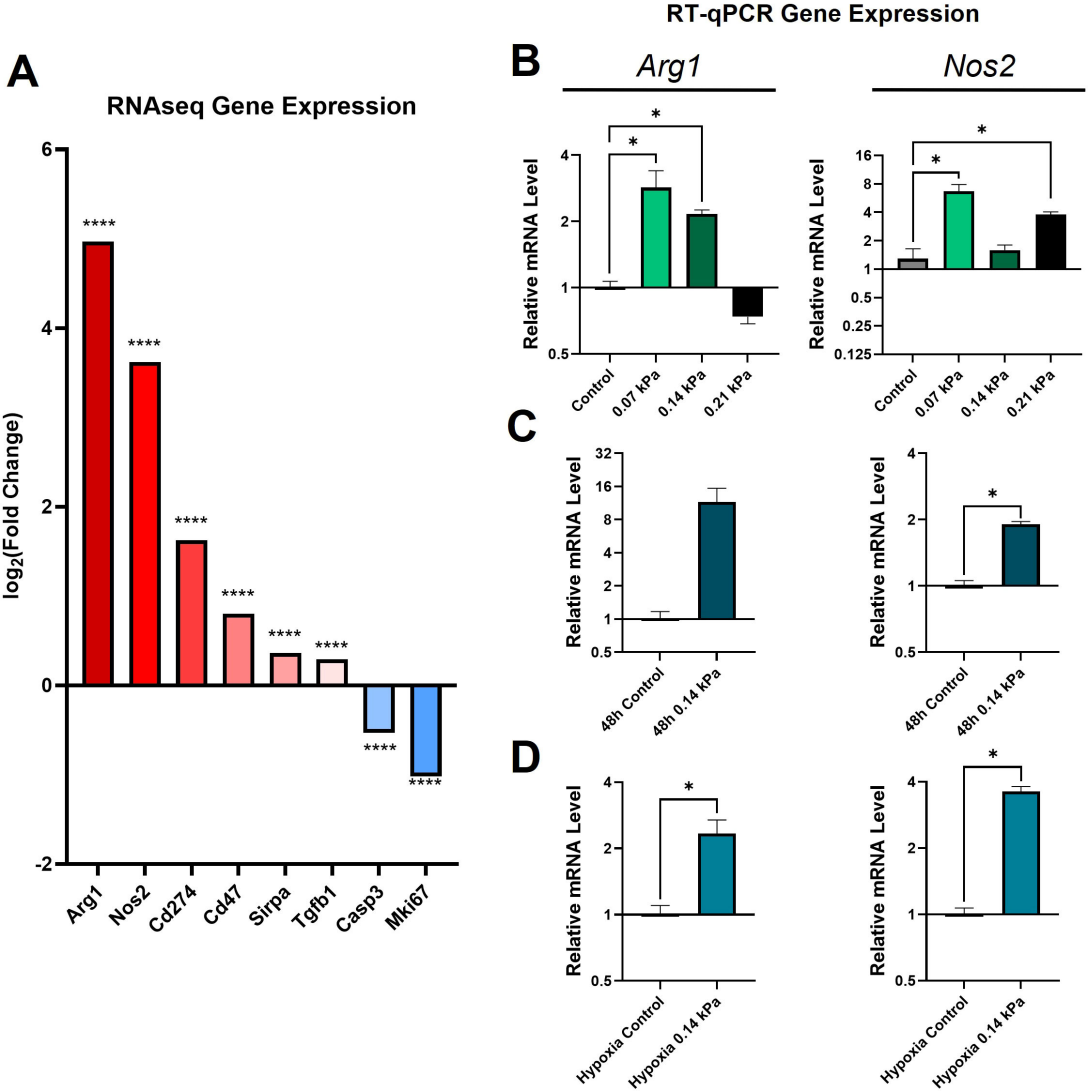
Compression causes upregulation of M1, M2, and immune escape-related genes largely independent of force magnitude/duration and oxygen tension. **(A)** Log_2_(fold change) of genes of interest based on RNA sequencing data. ****padj ≤ 0.0001. RT-qPCR quantification of *Arg1* and *Nos2* expression under various magnitudes of solid stress **(B)**, increased duration of solid stress **(C)**, and under simultaneous compression and hypoxia **(D)**. * p = statistically significant, adjusted for multiple comparisons.

We next obtained relative expression estimates of specific genes of interest from the sequencing data (**Figure 4A**). *Arg1* and *Nos2* (canonical markers of pro- and anti-inflammatory polarization, respectively) were both significantly upregulated. *Cd274* (encoding PD-L1), *Cd47*, and *Sirpa*, all implicated in tumor immune escape (73, 74), were also all significantly upregulated. However, both *Casp3* and *Mki67* were downregulated.

To determine whether the response to compression varies with compression magnitude, we applied 0.07, 0.14, and 0.21 kPa of compression to macrophages, corresponding to 0.5x, 1x, and 1.5x the original magnitude. We performed RT-qPCR analysis of *Arg1* and *Nos2*, as representatives of the pro-and anti-inflammatory states. There was a general trend towards increased *Arg1* and *Nos2* with various compression conditions (**Figure 4B**). Interestingly, the largest increase in *Arg1* occurred in the lowest compression group, showing a 2.9-fold increase under 0.07 kPa of compression. However, at 0.21 kPa, there was a non-significant decrease in RT-qPCR quantification of *Arg1*. *Nos2* was significantly upregulated in the 0.07 kPa and 0.21 kPa conditions by 6.7 and 1.6-fold, respectively, with a non-significant slight increase with 0.014 kPa. After 48 hours of 0.14 kPa compression, *Arg1* had a large, but not statistically significant, 11.6-fold increase, while *Nos2* had a statistically significant 1.9-fold increase (**Figure 4C**). To determine whether this effect would remain under hypoxia, such as what is observed in solid tumors, we applied 0.14 kPa of compression to macrophages for 24 hours cultured in 1% O_2_ (**Figure 4D**). Both *Arg1* and *Nos2* showed statistically significant increases of 2.3-fold and 3.6-fold, respectively. While the biological variability of the macrophage transcriptional response to compression hindered the achievement of statistical significance in some conditions, these data generally demonstrate an upregulation of canonical markers of both pro- and anti-inflammatory macrophage phenotypes under various compression conditions.

We also performed Western blot analysis of relevant proteins, including IL-1β, MMP9, STAT3, and phosphorylated STAT3 (**Supplementary Figure S4A-D**). We observed no clear difference in protein abundance between compressed and control conditions for these targets. This may be due to post-translational regulation, such as the cleavage step required to convert pro-IL-1β to active IL-1β (75). The enrichment of the JAK/STAT3/IL-6 and ECM regulation pathways may also be driven by other upstream or downstream elements of the pathway, such as various upregulated interleukins and members of the tumor necrosis factor receptor superfamily (TNFRSF) and might not be reflected in individual protein abundance.

### Compression induces increased average fluorescence lifetime associated with pro-tumor metabolism

**Figure 5:**
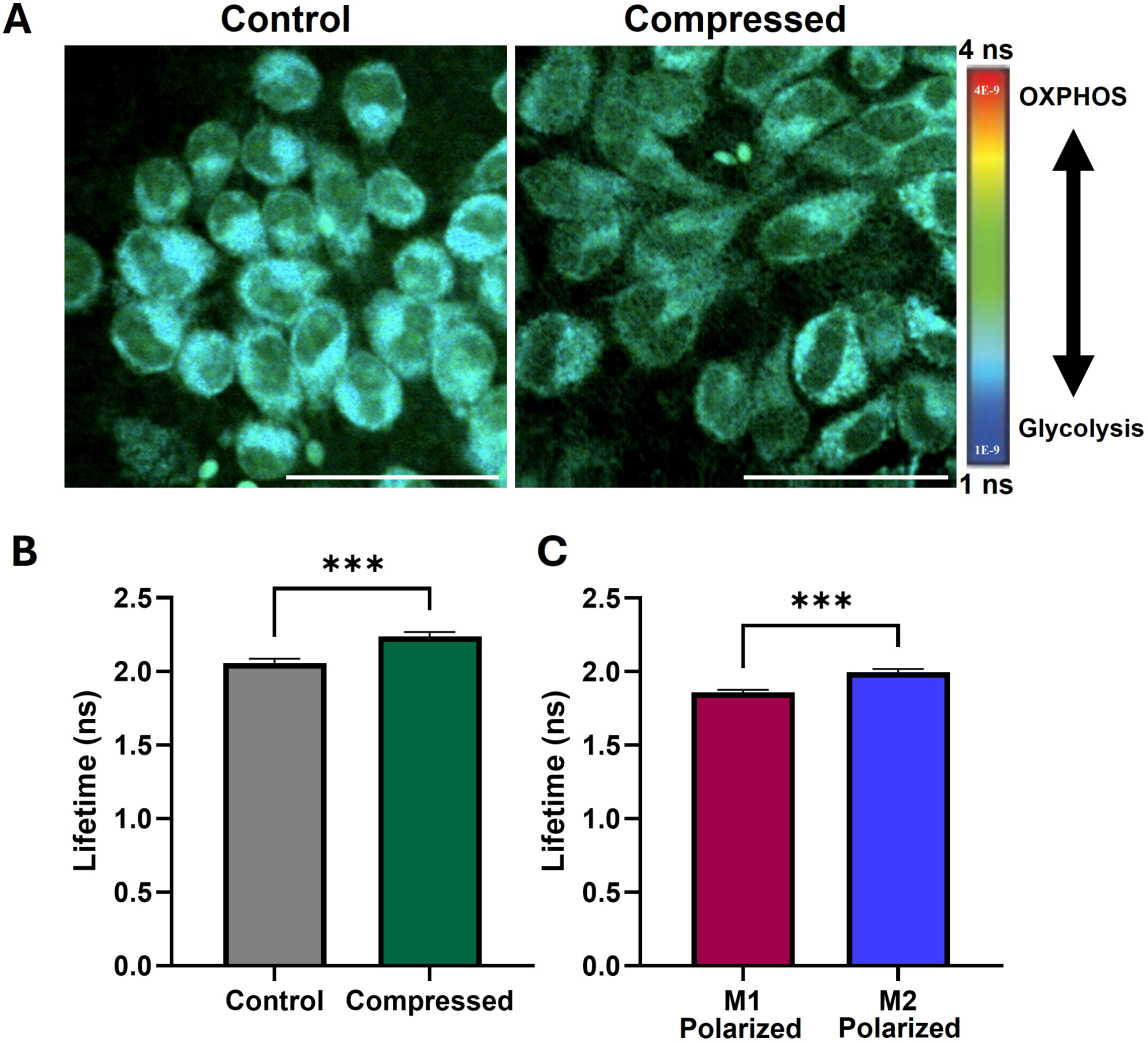
Compression increases fluorescence lifetime of macrophages, indicating increased oxidative phosphorylation and pro-tumor metabolism. **(A)** Fluorescence lifetime images of uncompressed and compressed macrophages. Cooler colors indicate lower fluorescence lifetime and increased reliance on glycolysis, and warmer colors indicate increased lifetime and increased reliance on oxidative phosphorylation (OXPHOS). Scale bar represents 50 µm. **(B)** Mean fluorescence lifetime in compressed versus control macrophages. **(C)** Mean fluorescence lifetime in macrophages treated with M1 and M2 polarizing cytokines, confirming predicted upward shift with M2-polarization observed in literature. ***p ≤ 0.001.

NAD(P)H, a metabolic coenzyme, has an autologous fluorescence signal with variable fluorescence lifetime depending on whether it is free or bound to a protein (76). NAD(P)H in the bound state has a longer fluorescence lifetime and indicates oxidative phosphorylation, while free NAD(P)H is involved in glycolysis. Because NAD(P)H is the predominant intracellular auto-fluorescent molecule, measuring the average fluorescence lifetime of a cell indicates whether it relies more on oxidative phosphorylation (OXPHOS) or glycolysis. Macrophages are reported to have a significant shift in lifetime with polarization, where M1-like macrophages tend to have a lower lifetime and increased reliance on glycolysis while M2-like macrophages have an increased lifetime and rely on oxidative phosphorylation instead (77). We successfully generated fluorescence lifetime microscopy (FLIM) images of compressed and uncompressed macrophages (**Figure 5A**). We then obtained the average fluorescence lifetime of the cell area for each image. We observe that compressed macrophages significantly increase average fluorescence lifetime compared to uncompressed controls, a shift which is consistent with a more anti-inflammatory, M2-like polarization (**Figure 5B**). We confirmed that macrophages induced to have an anti-inflammatory, M2-like polarization (treated with IL-4) had a higher average lifetime than pro-inflammatory M1-like macrophages (treated with LPS and IFN-γ), consistent with prior reports (77) (**Figure 5C**).

### Compression promotes increases in functional markers of pro- and anti-inflammatory states

**Figure 6:**
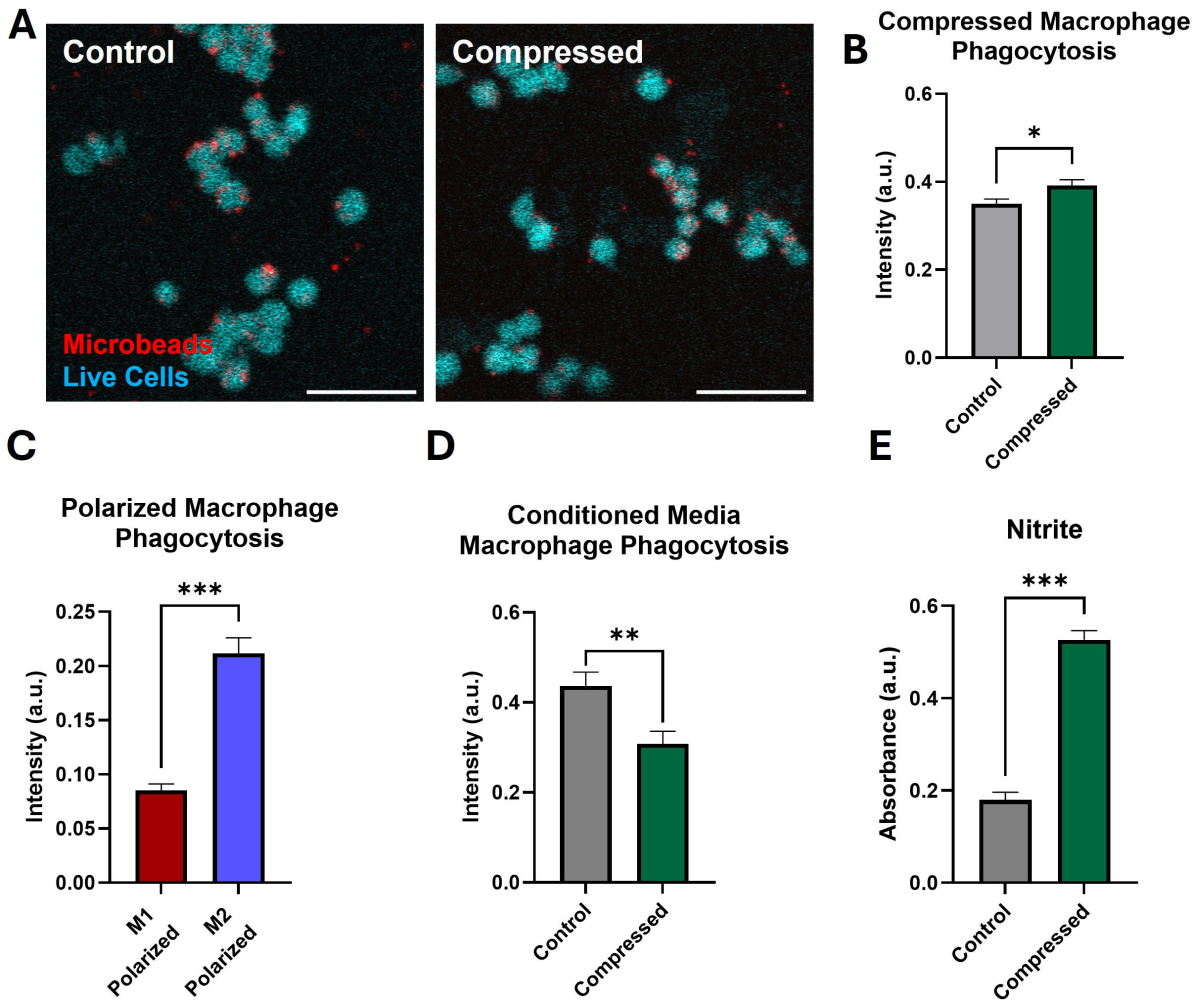
Macrophages under compression have increased phagocytic capacity and nitric oxide production. **(A)** Representative images of macrophages (cyan) and fluorescent microbeads (red). After 24 hours of compression, macrophages were incubated with fluorescent microbeads, internalizing them based on phagocytic capacity. Scale bar represents 50 µm. **(B)** Quantification of intensity of intracellular fluorescent microbeads phagocytosed after compression as a metric of the overall phagocytic capacity. **(C)** Quantification of microbead uptake by M1-like or M2-like macrophages treated with treated with LPS and IFN-γ or IL-4, respectively. **(D)** Quantification of microbead uptake by macrophages cultured with conditioned media from control or compressed macrophages. **(E)** Quantification of nitrite (indicative of NOS) released by macrophages after 48h of compression. *p ≤ 0.05, **p ≤ 0.01, ***p ≤ 0.001.

Finally, to determine how compression alters macrophage function, we performed a phagocytosis assay on macrophages co-cultured with fluorescent microbeads immediately after 24 hours of compression. We observed a significant increase in microbead uptake in compressed macrophages (**Figure 6A, B**). Increased phagocytic capacity is reported to be associated with the M2-like polarization state, in line with our observation of increased phagocytosis by M2-polarized macrophages compared to M1-polarized (**Figure 6C**) (78). To determine whether secreted signals by compressed macrophages influenced uncompressed cells, we treated macrophages grown in standard culture conditions with conditioned media from compressed or uncompressed macrophages. We found a significant decrease in phagocytosis by macrophages treated with media conditioned by compressed macrophages, indicating potentially competing primary and secondary effects of compression (**Figure 6D**). Secreted nitric oxide (NOS), a mediator of macrophage cytotoxic activity, was measured via a Griess assay of compressed macrophage-conditioned media. After 48 hours of compression, there was a statistically significant increase in the nitrite, a derivative of secreted NOS, in conditioned media (**Figure 6E**). While NOS production is a canonically M1-like marker, its secretion by TAMs may promote treatment resistance (79).

To determine the indirect effect that compression may have on co-cultured macrophages and GBM cells, we conducted co-culture compression experiments using the CT-2A murine GBM cell line. We compressed a layer of murine GBM cells on top of the transwell membrane, and cultured macrophages in the bottom of the same well, such that the macrophages were not in physical contact with the GBM cells and were not compressed but were exposed to GBM-derived secreted factors (**Supplementary Figure S5**). This allowed for the specific assessment of whether compression of one cell type causes changes in paracrine signaling that influences the other cell type. We observed no significant change in macrophage expression of *Arg1, Nos2*, *Il1b* or *Serpinb2* (**Supplementary Figure S5A**). Likewise, when GBM cells were seeded in the bottom of the plate with macrophages compressed on top (**Supplementary Figure S5B**), we observed no significant change in GBM cell *Ki67, Casp3*, or *CD47* expression. These results suggest that the direct effect of compression on cells may be more important than the indirect effect through cell-cell paracrine signaling.

## Discussion

Here, we establish a clear relationship between the mechanical TME and its immune residents. The direct contribution of macrophages to tumor progression, immune escape, and patient outcome has been extensively studied and is well-reviewed elsewhere (2, 5, 6, 80). Macrophages are known to respond to their physical environment, including matrix stiffness and topography, cyclic tension or compression, and shear stress (25, 81). However, studies on compressive solid stress on macrophages have generally been limited to the context of bone or orthodontic intervention, so the magnitudes of solid stress and the environments in which they are studied differ drastically from those found in the TME (33, 34, 36). Our custom compression system is designed to apply mild, chronic compressive stress (0.14 kPa), within the range of what has been observed in murine and human GBM tumors (0.1 to 0.2 kPa) (14–17). This study is the first of its kind to study chronic compressive stress on macrophages in a custom compression system designed to mimic the mechanical stress in the GBM TME.

**Figure 7:**
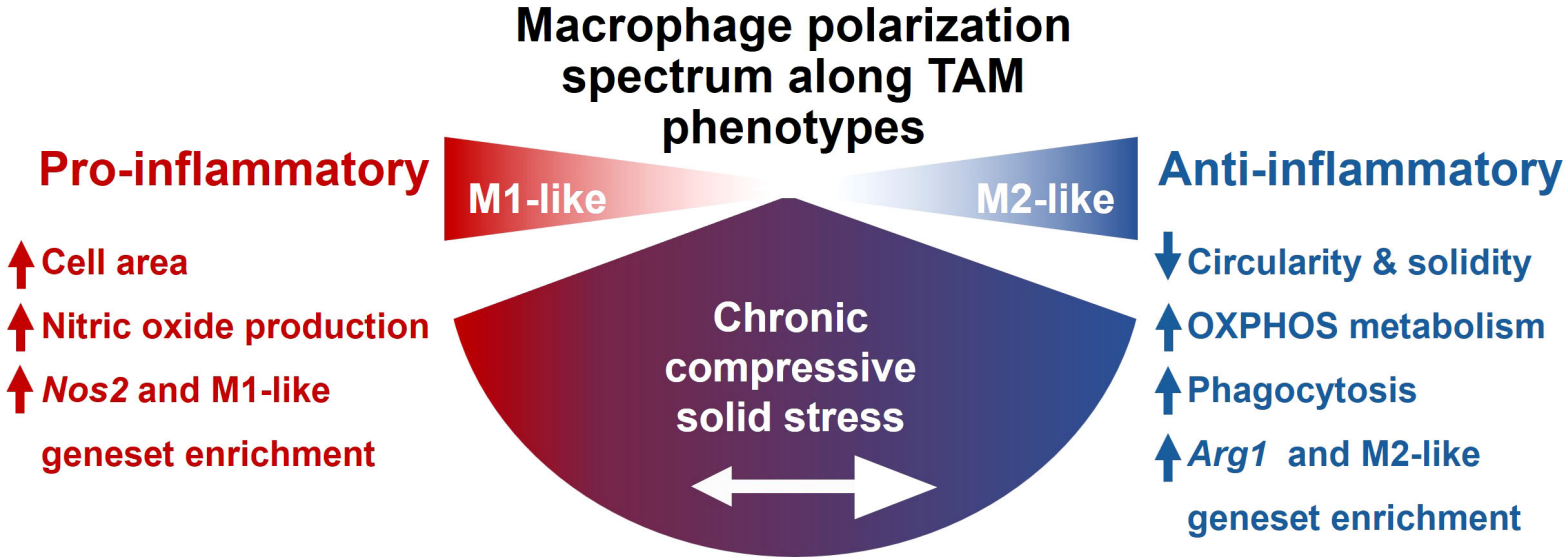
Overview of the effect of compression on macrophages. Compression causes macrophages to adopt both pro- and anti-inflammatory associated traits, with the net effect of promoting an immunosuppressive, tumor-associated and glioma-associated macrophage phenotype.

For the various metrics we applied, compression caused alterations resembling both pro- and anti-inflammatory polarization states (**Figure 7**). For example, increased macrophage size, seen under compression, is a typical characteristic of pro-inflammatory macrophages (48). However, the more irregular cell shape (characterized by circularity and solidity) is more commonly associated with the anti-inflammatory phenotype (48). Macrophage morphology is not only an indicator of polarization but can also directly impact it as part of a two-way interaction. For example, macrophages forced to adopt an elongated shape have altered polarization and sensitivity to pro-inflammatory stimuli (49). In our system, the unconfined, uniaxial compression may have physically forced the cells to spread thinner, resulting in the observed increased area. This forced change in shape may itself be one mechanism by which compression drives changes in macrophage phenotype. Interestingly, Hippo-related pathways were not significantly altered by compression, potentially explaining the lack of profound alterations to YAP/TAZ translocation in **Supplementary Figure S1**.

Functionally, compressed macrophages were more phagocytic, in line with gene expression data, and released more nitric oxide into their media. Elements of the IL6/JAK/STAT3 signal pathway were also upregulated, which are associated with worse prognosis in solid tumors and important in regulating macrophage response to stimuli (58, 82). Compressed macrophages also upregulated ECM remodeling-associated genes, including several MMPs. MMP9, a prominent hub on the protein interaction map, promotes cancer cell invasion and angiogenesis and is associated with poor patient prognosis in solid tumors (65). The impact of this functional mechano-immunological reprogramming remains to be fully elucidated in GBM models.

Based on gene ontology analysis, the biological and molecular processes upregulated under compression were largely related to chemotaxis and cytokine signaling, while many of the most downregulated processes were involved in cell replication. Visualizing these two processes in a protein-protein interaction map highlighted IL-1β (which was highly upregulated under compression) as one of the central hubs of interaction for both. While this is considered a pro-inflammatory macrophage gene in healthy contexts, the net effect of TAM expression of IL-1β is decreased immune response efficacy, treatment resistance, and disease recurrence in cancer (54). IL-1β participates in a feedback loop in several human cancers, promoting tumor immune escape via increased PD-L1 expression, the secretion of macrophage-recruiting proteins, the acquisition of malignant characteristics by tumor cells, and worse patient prognosis (83–86). In GBM in particular, macrophage-derived IL-1β promotes edema, induces GBM tumor growth, promotes cancer stem cell phenotype, and leads to further immune reprogramming by GBM cells including macrophage polarization and recruitment (55, 87–90). The induction of IL-1β production by macrophages is attributed to factors secreted by tumor cells, but here we show that compression alone, in the absence of any tumor-derived biochemical signals, causes an increase in *Il1b* gene expression. IL-1β may therefore be an important intermediary between the pathological mechanical microenvironment and macrophage immune reprogramming.

We note that compression caused a simultaneous decrease in pathways associated with cell cycling and proliferation, and an increase in genes associated with apoptosis. Together, these would suggest that compression may induce a decrease in the macrophage population in a closed system. Weak macrophage proliferation within tumors is one of the biggest hurdles for engineered macrophage-based therapies, such as CAR-macrophages (91). However, the concurrent increase in pathways related to migration and chemotaxis suggests that an open system, such as a tumor *in vivo*, accumulates macrophages in response to compression via recruitment from the bloodstream and migration into the tumor. Compressed macrophages are not only potentially more migratory themselves, but their upregulation of monocyte chemoattractants such as CCL2 and CCL5 (constituents of the migration-associated geneset) indicate that they could play an active role in the direct recruitment of more macrophages from the bloodstream, as TAMs are observed to do in solid tumors (92).

Macrophage polarization did not neatly align with either the M1 or M2 side of the canonical polarization spectrum. As has been observed in gliomas, compressed macrophages upregulated both M1-like and M2-like genes, and traditional polarization markers do not necessarily correlate with function in the context of the TME (62, 93). The upregulation of the “Inflam-TAM 2” geneset (**Figure 3CB**) is not representative of a healthy, effective inflammatory response, but rather of inflammatory states in the tumor microenvironment (66). The concurrent upregulation of genes associated with M1-like and M2-like polarization is consistent with our observations of macrophages in 3D confinement and 3D uniaxial compression (32). As illustrated by these data, the traditional, simplified M1-M2 polarization spectrum cannot fully capture complex macrophage states. Macrophages have been observed to concurrently express M1 and M2 markers in both health and disease, and both sides of the polarization spectrum can be co-opted in the tumor microenvironment to serve a pro-tumor function (4), including in glioma/glioblastoma, as we and others have shown (21, 94).

Here, we leveraged FLIM to obtain high-sensitivity, *in situ* metabolic measurements without the need for stains or probes. FLIM has been shown to correlate with both mass spectrometry and flux analysis, and has the added advantage of providing high-resolution spatial metabolism data (95). M2-like macrophages have been reported to have a longer average fluorescence lifetime than M1-like macrophages, a characteristic that can help distinguish the two with relatively high accuracy (77, 96). The increased fluorescence lifetime of compressed macrophages thus suggests a shift towards a more M2-like metabolic state, in line with our M1-like and M2-like polarized macrophage controls (**Figure 5B, C**). The increase in fluorescence lifetime is expected to correspond with more reliance on oxidative phosphorylation, and less on glycolysis (97). As noted by others, average fluorescence lifetime alone is influenced by factors other than oxidative or glycolytic cell state, so more comprehensive FLIM analysis or other metabolic assays (such as real-time metabolic flux assays) will be required to more fully understand cellular metabolic state (98). Interestingly, hypoxic conditions are expected to be accompanied by a decrease in cell fluorescence lifetime (99, 100), but compressed macrophages simultaneously upregulated hypoxia genes and had increased average fluorescence lifetime. Macrophages polarized to an M1-like state by interferon-gamma rely on increased glycolysis, which allows for Hif-1a, IL-1b, and nitric oxide production and JAK/STAT signaling - all consistent with our gene expression and media analyses (38). Together, these results demonstrate the complexity of macrophage polarization and metabolism and highlight the need for further mechanistic studies linking the two in the context of chronic compression.

Targeting myeloid cells has shown great promise as part of a combinatorial approach, enhancing the effect of other strategies to alter the TME. For example, macrophages are essential for the full beneficial effect of dual inhibition of Ang-2 and VEGF in murine GBM (10). Infiltrating macrophages engage in CCR2-CCL2 signaling in GBM, which is reported to be associated with reduced overall survival (21, 101, 102). Targeting myeloid cells in the GBM TME by CCR2 inhibition is sufficient to remove the barrier to ICB efficacy and extends survival in murine GBM models (102). Reprogrammed macrophages may also serve a as powerful therapeutic tool, as pharmacologically normalizing the tumor vasculature results in improved survival mediated by reprogrammed tumor-resident macrophages (10). This strategy is advantageous because it can target myeloid cells in the bone marrow and therefore does not need to cross the BBB to have effect. Targeting solid stress in the TME will also interrupt the immune-mechanical feedback loop. Reducing any of the solid components of the tumor (tumor cells, stromal cells, or ECM) causes a reduction in solid stress (19). Losartan, an angiotensin system inhibitor, can help control ICB-related adverse events and its use is associated with improved overall survival in both murine and human GBM (21, 103). This may be due to its inhibition of collagen/hyaluronan synthesis, CCR2 signaling, and monocyte recruitment, potentially simultaneously impacting both TAMs and the mechanical TME (104, 105).

In conclusion, we have demonstrated that chronic mechanical compression alone can drive macrophages towards a complex phenotype largely resembling that seen in GBM and other cancers. We previously established that macrophages themselves can contribute to the generation of solid stress (32). This work suggests that there may be a positive immunomechanical feedback loop between macrophages and solid stress, wherein macrophages may both generate and respond to solid stress in a manner that contributes to immune suppression and tumor progression. This cycle represents a promising therapeutic target, as reducing solid stress, macrophage sensing and response to it, or macrophage contribution to it, could break the cycle and normalize the tumor immune microenvironment.

## Data Availability

The original RNA sequencing data has been uploaded to NCBI GEO, under the accession number GSE304867 and can be downloaded at the following address: https://www.ncbi.nlm.nih.gov/geo/query/acc.cgi?acc=GSE304867.
