# Supplementary figures and images for: Chronic compression induces transcriptional, metabolic, and functional state changes in macrophages that recapitulate tumor-associated phenotypes
Source: Front Immunol. 2025 Dec 9;16:1626024. doi: 10.3389/fimmu.2025.1626024 (PMC12722438; doi:10.3389/fimmu.2025.1626024)

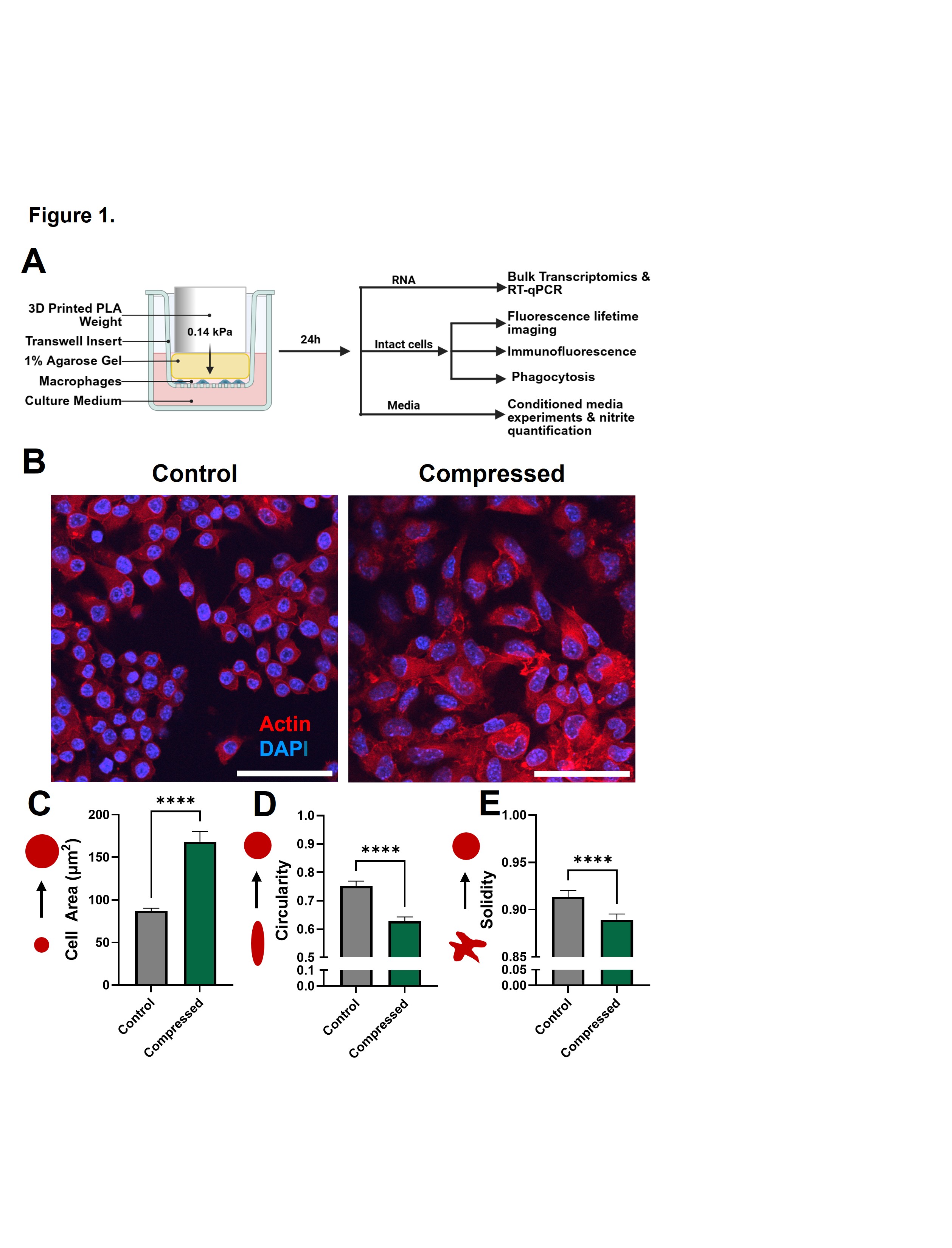

Supplement: Supplementary file 1 [file DataSheet1.zip › AllFigures_111825/MainFigures_111825/Burchett_FrontImmunol_111825_Figure1.jpg]

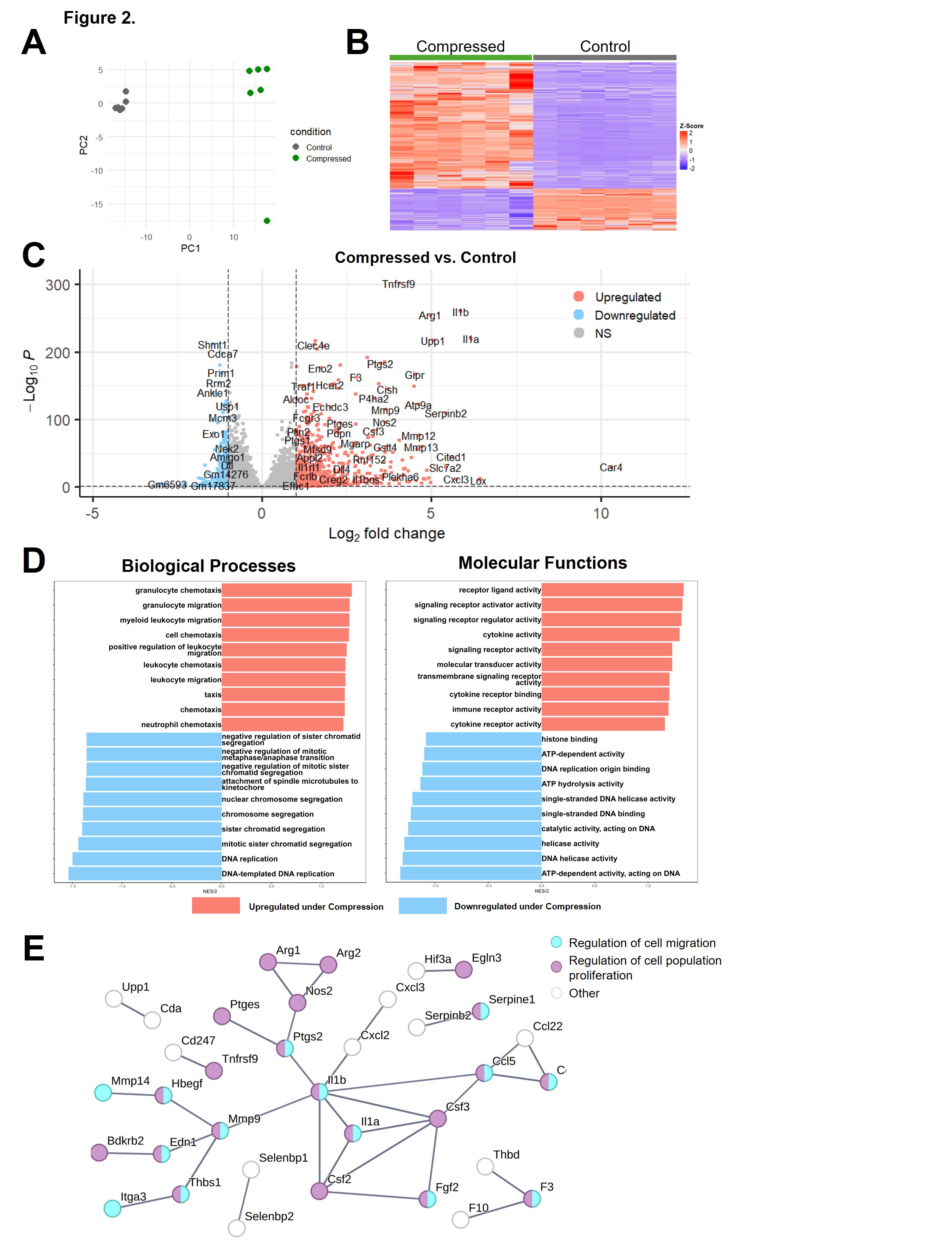

Supplement: Supplementary file 1 [file DataSheet1.zip › AllFigures_111825/MainFigures_111825/Burchett_FrontImmunol_111825_Figure2.jpg]

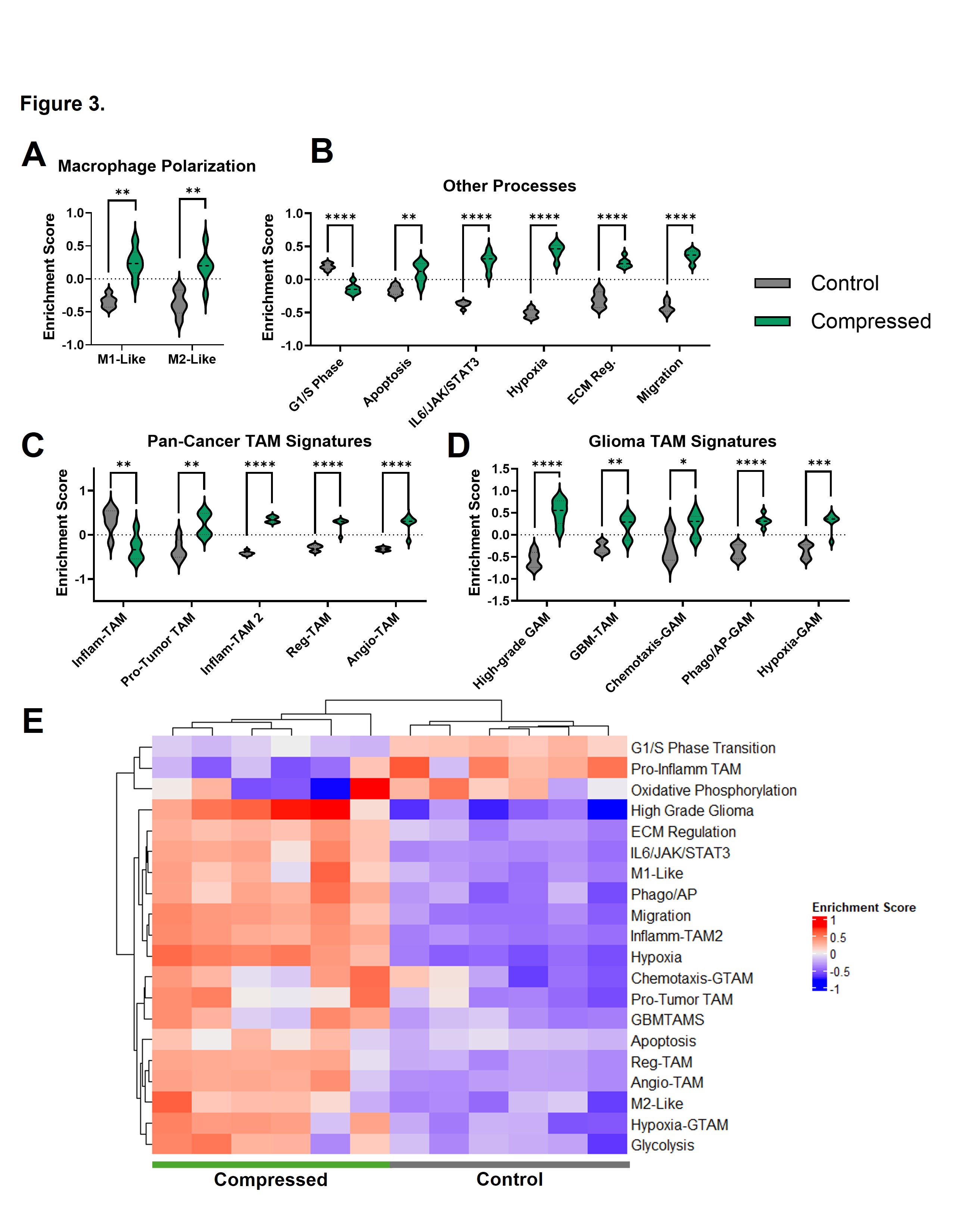

Supplement: Supplementary file 1 [file DataSheet1.zip › AllFigures_111825/MainFigures_111825/Burchett_FrontImmunol_111825_Figure3.jpg]

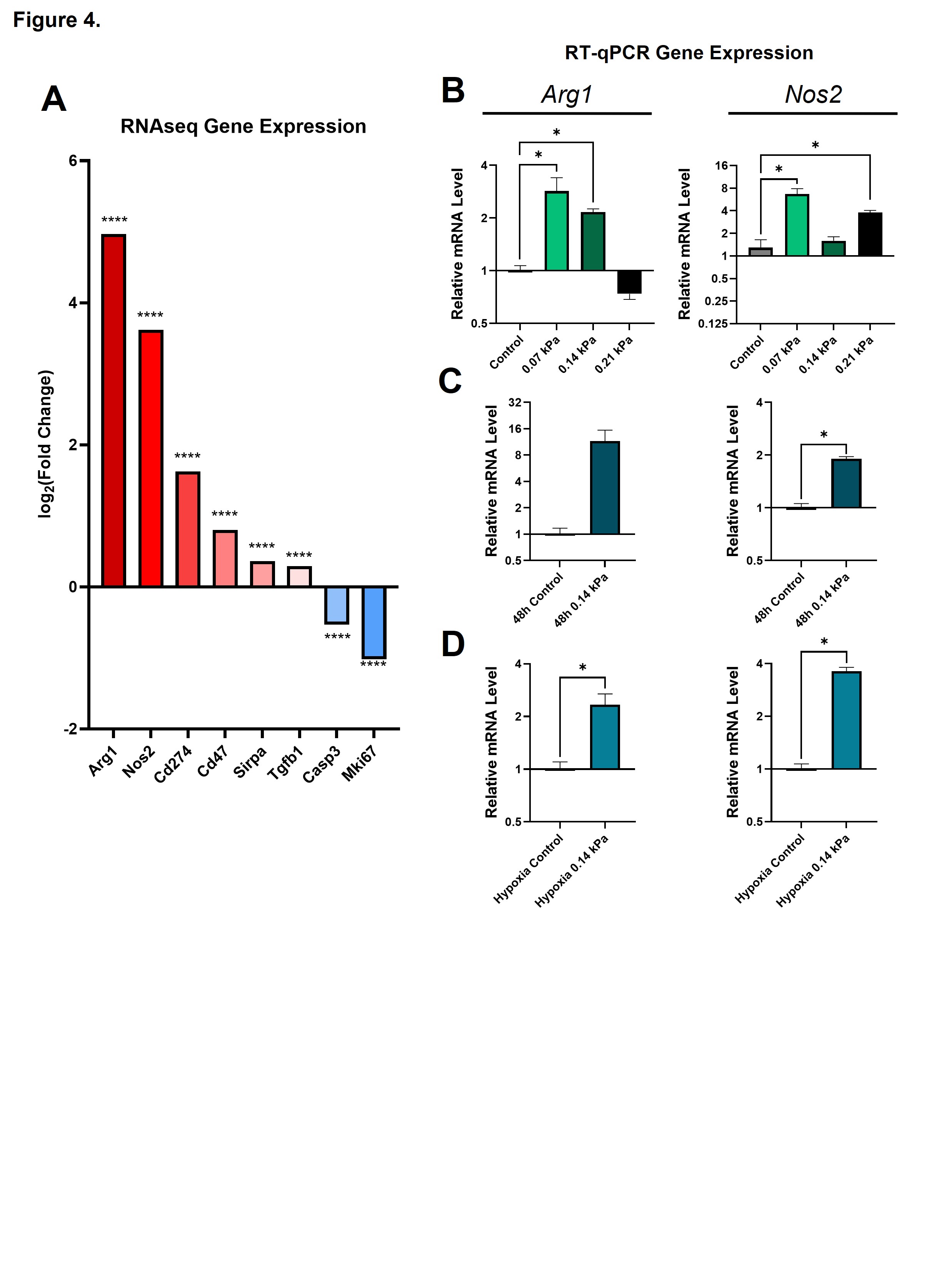

Supplement: Supplementary file 1 [file DataSheet1.zip › AllFigures_111825/MainFigures_111825/Burchett_FrontImmunol_111825_Figure4.jpg]

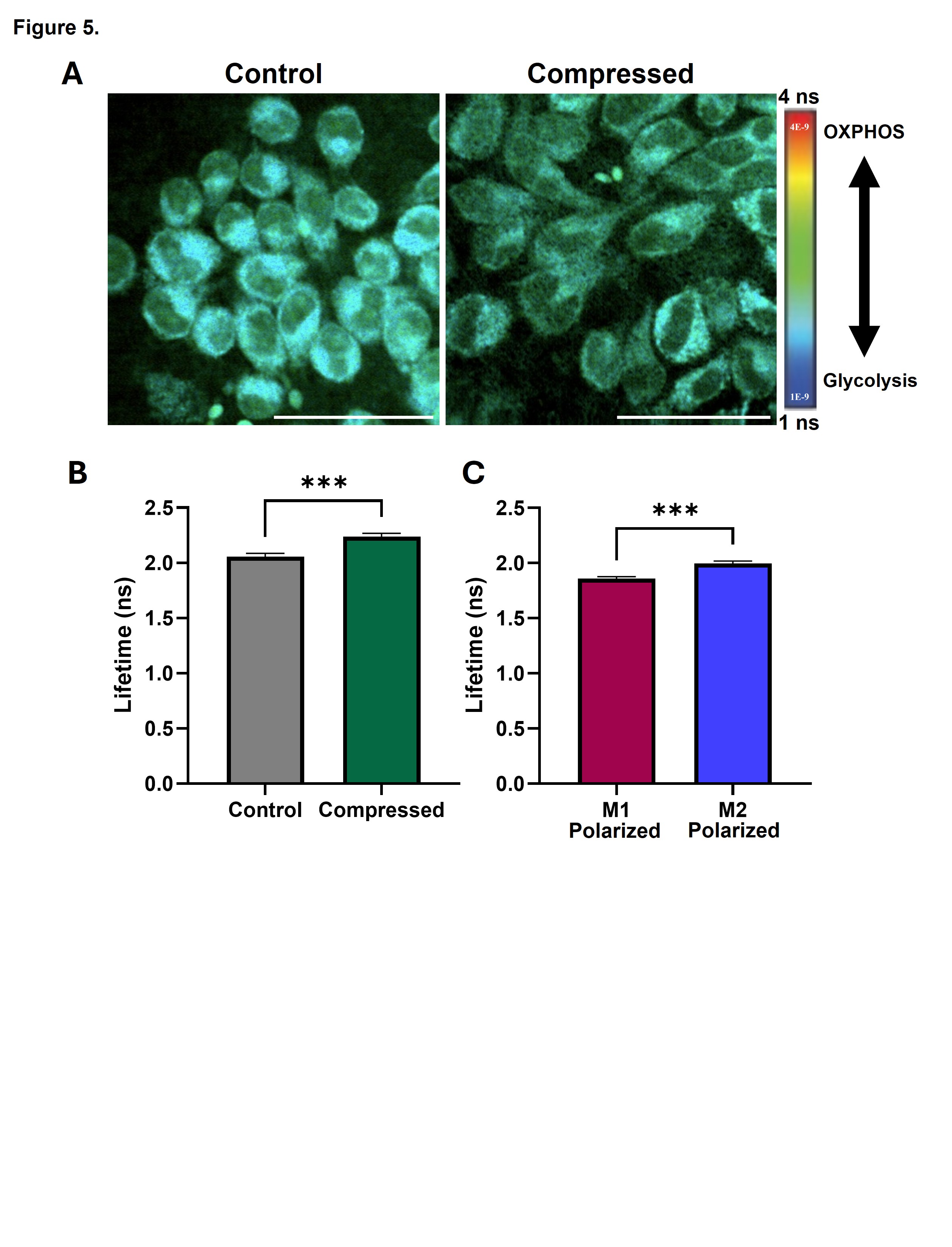

Supplement: Supplementary file 1 [file DataSheet1.zip › AllFigures_111825/MainFigures_111825/Burchett_FrontImmunol_111825_Figure5.jpg]

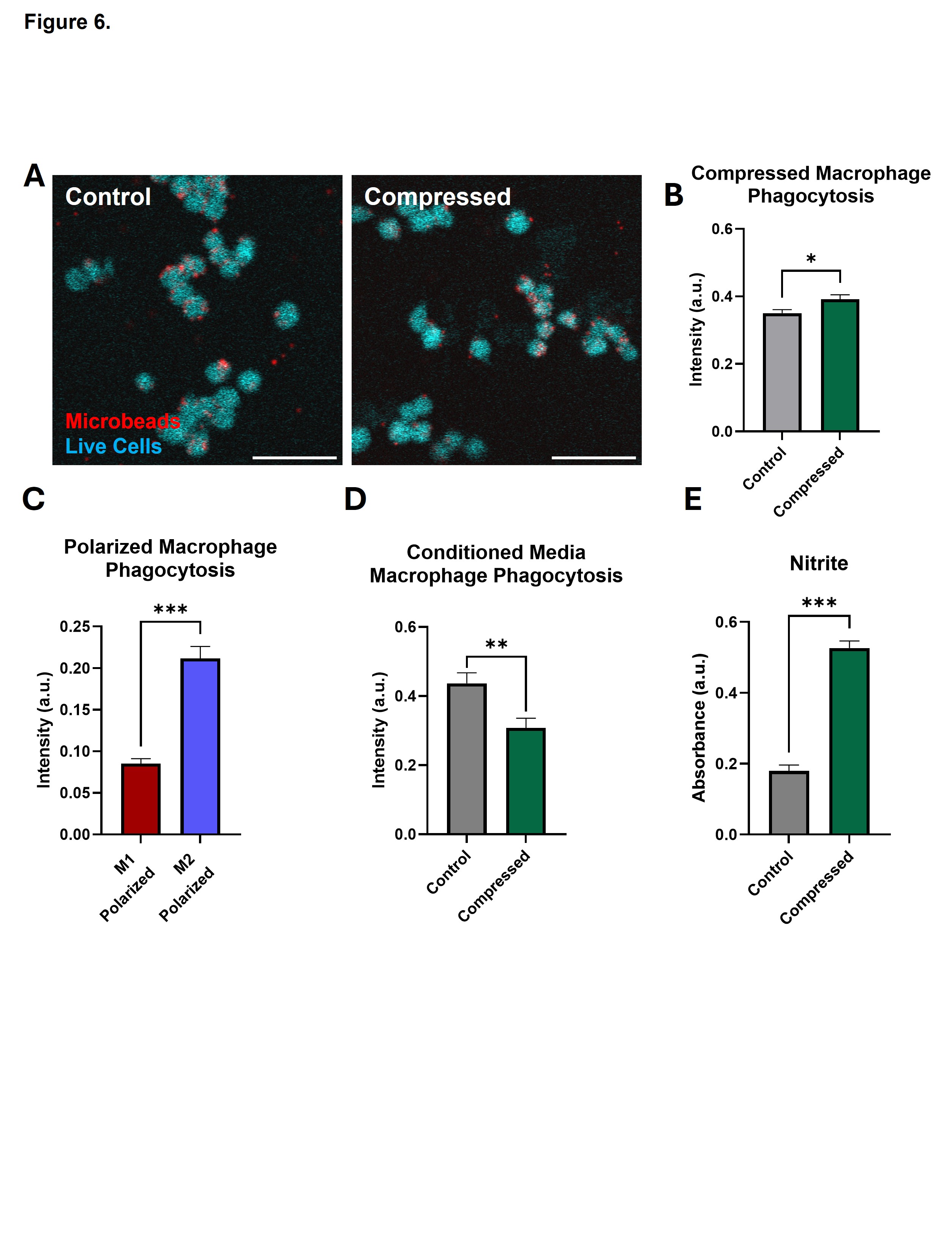

Supplement: Supplementary file 1 [file DataSheet1.zip › AllFigures_111825/MainFigures_111825/Burchett_FrontImmunol_111825_Figure6.jpg]

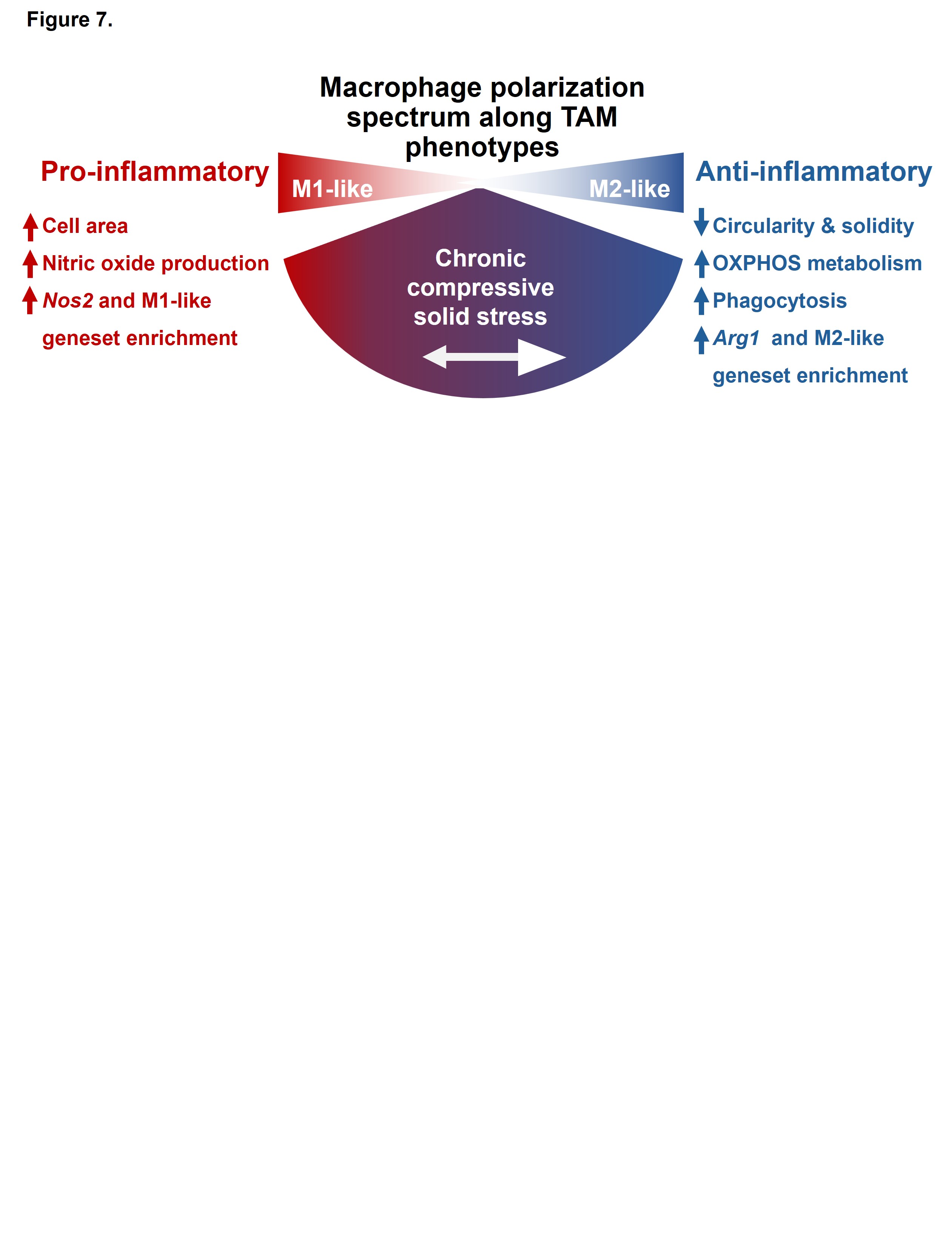

Supplement: Supplementary file 1 [file DataSheet1.zip › AllFigures_111825/MainFigures_111825/Burchett_FrontImmunol_111825_Figure7.jpg]

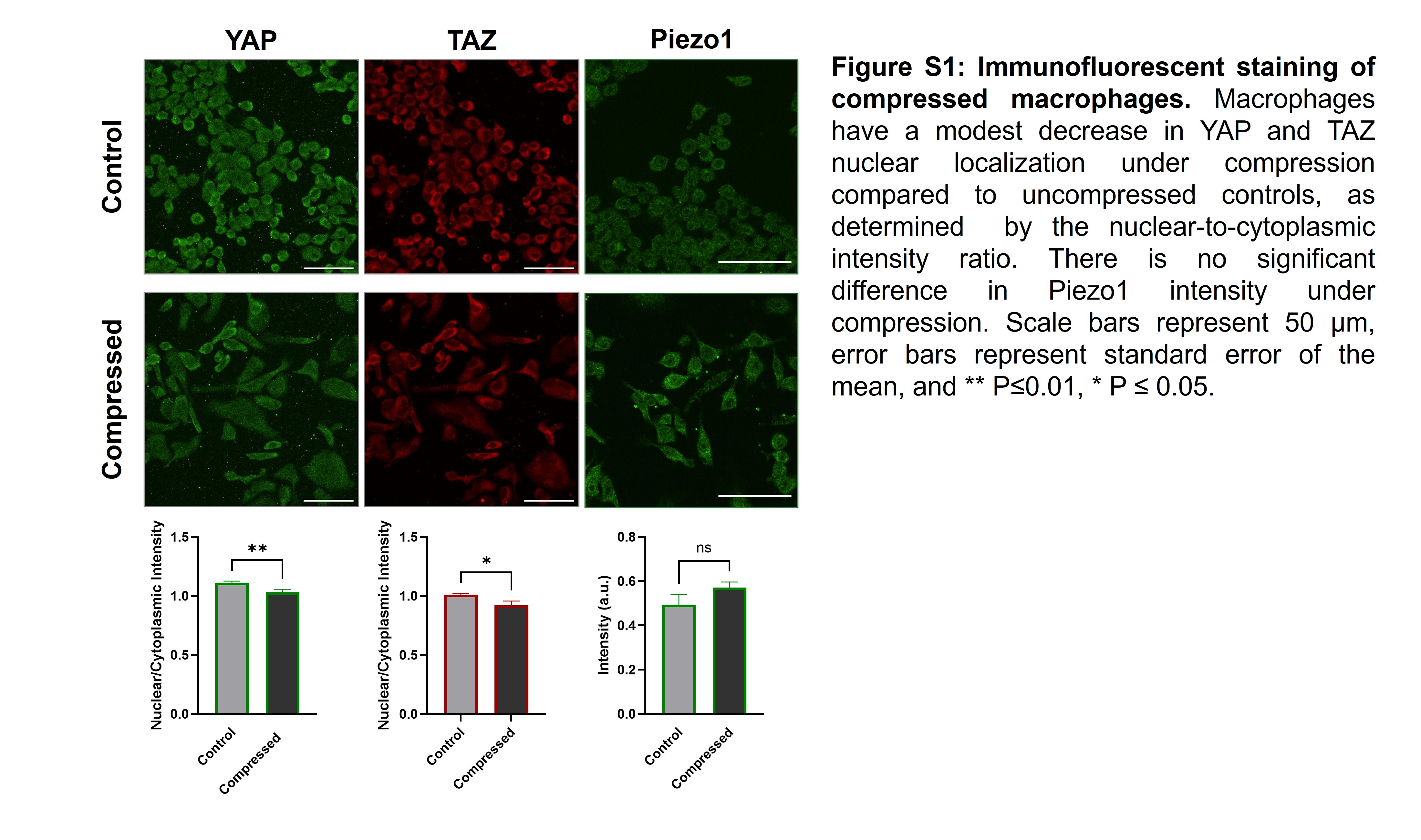

Supplement: Supplementary file 1 [file DataSheet1.zip › AllFigures_111825/SupplementaryFigures_111825/Burchett_FrontImmunol_111825_SupplementaryFigure_S1.jpg]

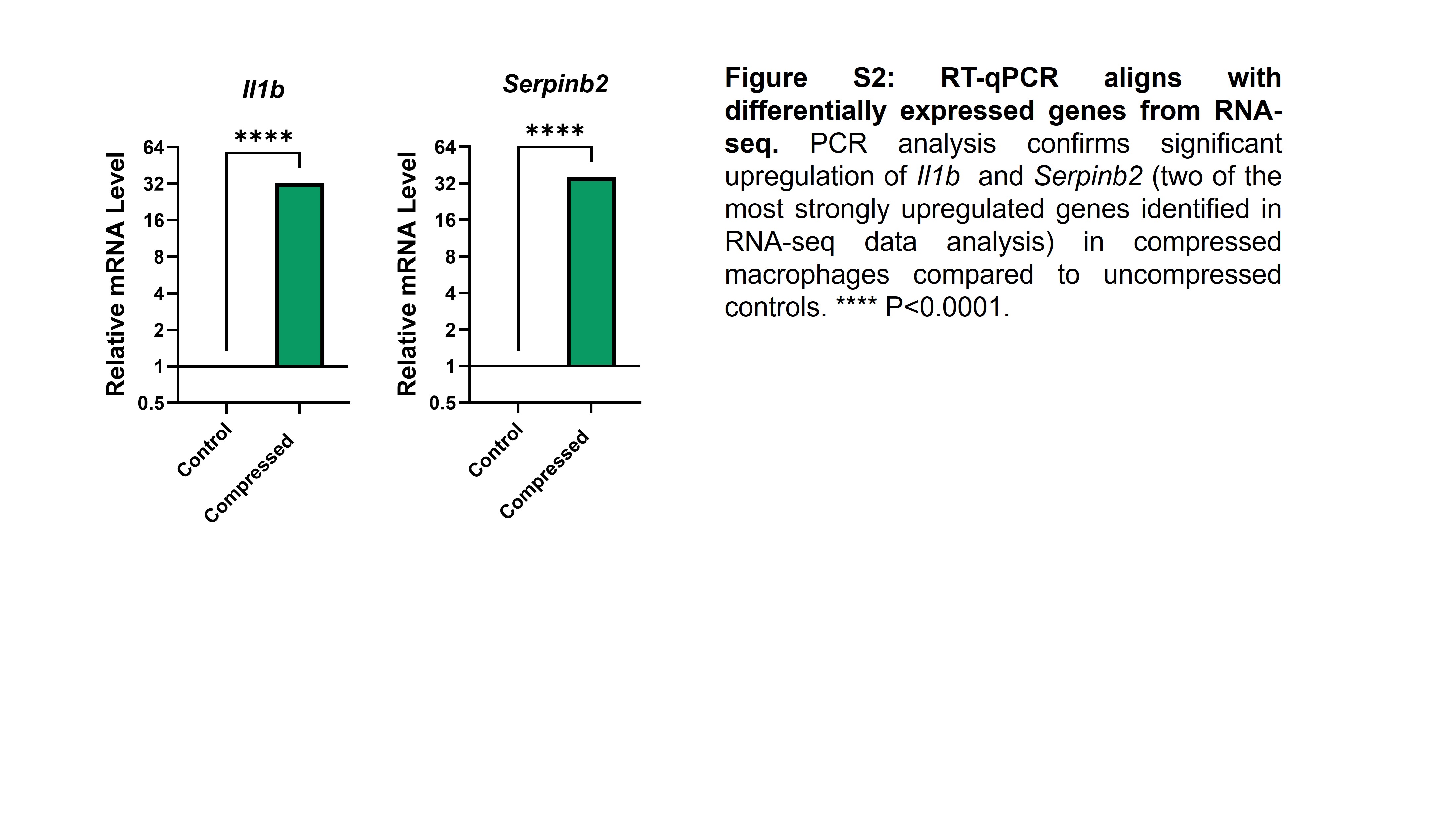

Supplement: Supplementary file 1 [file DataSheet1.zip › AllFigures_111825/SupplementaryFigures_111825/Burchett_FrontImmunol_111825_SupplementaryFigure_S2.jpg]

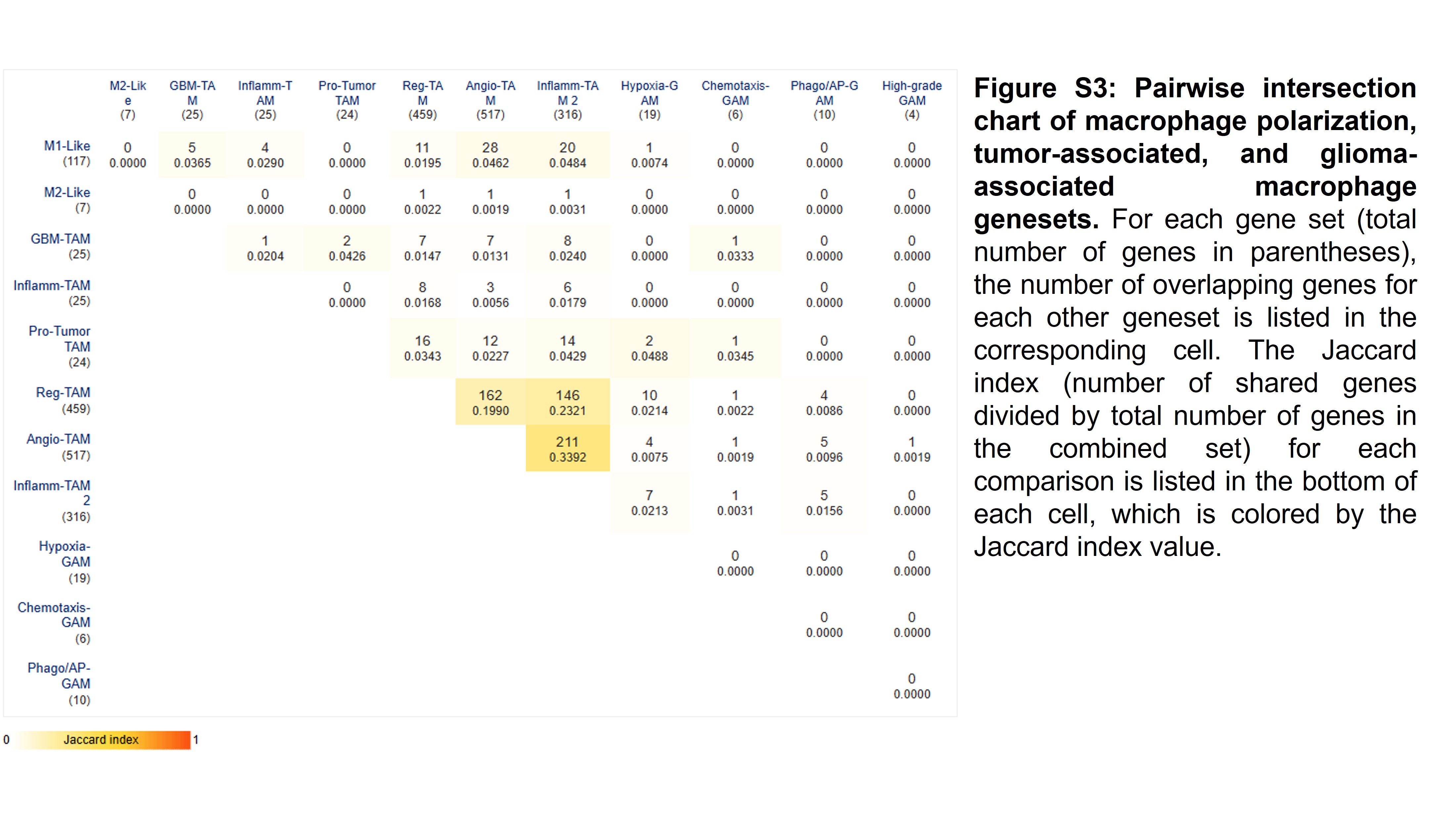

Supplement: Supplementary file 1 [file DataSheet1.zip › AllFigures_111825/SupplementaryFigures_111825/Burchett_FrontImmunol_111825_SupplementaryFigure_S3.jpg]

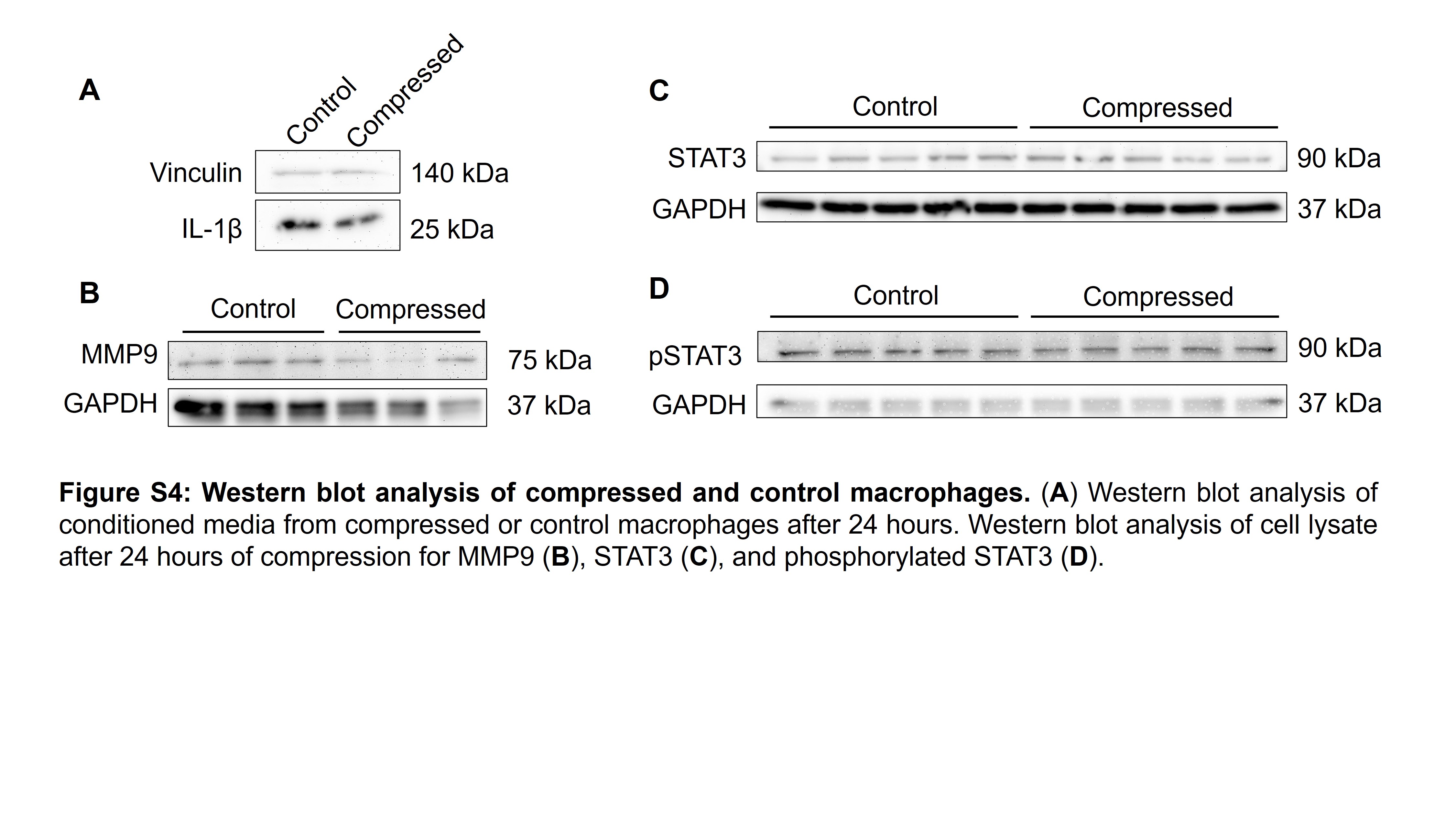

Supplement: Supplementary file 1 [file DataSheet1.zip › AllFigures_111825/SupplementaryFigures_111825/Burchett_FrontImmunol_111825_SupplementaryFigure_S4.jpg]

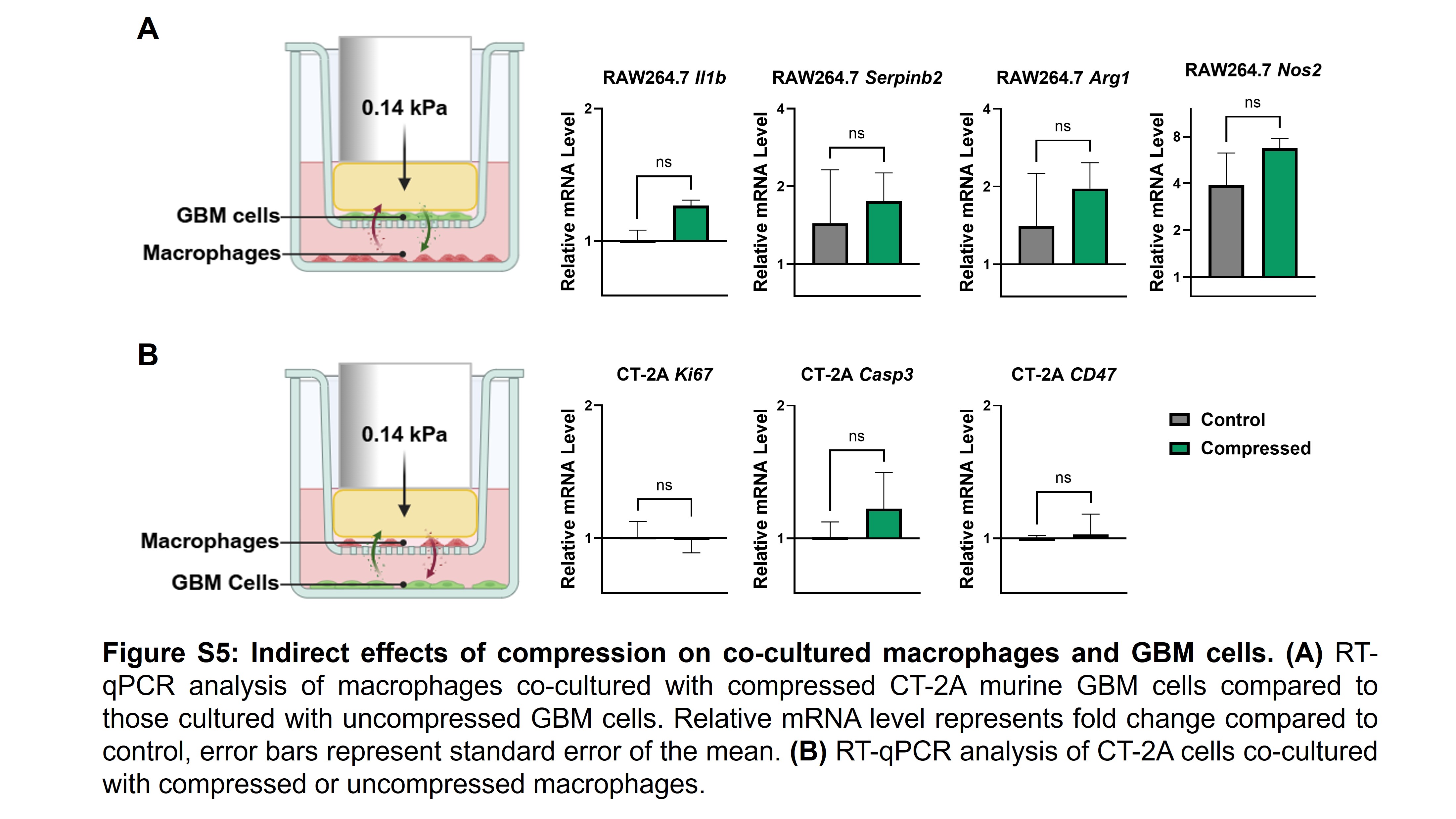

Supplement: Supplementary file 1 [file DataSheet1.zip › AllFigures_111825/SupplementaryFigures_111825/Burchett_FrontImmunol_111825_SupplementaryFigure_S5.jpg]
